# Supplementary material for: Assembly landscape for the bacterial large ribosomal subunit
Source: Nat Commun. 2023 Aug 26;14:5220. doi: 10.1038/s41467-023-40859-w (PMC10460392; doi:10.1038/s41467-023-40859-w)
Supplement: Supplementary file 1 — Supplementary Information [file 41467_2023_40859_MOESM1_ESM.docx]

**Supplementary Information**

**Table of contents**

**Supplementary Fig. 1-28**

**Supplementary Table 1-5**

**Supplementary Discussion**

**1 Characterization of the strain and intermediate peaks**

1-1 pre50S intermediate accumulated in ∆*deaD* strain upon cold shock

1-2 Mass spectrometry methods

1-2-1 Doubling time of WT and ∆*deaD* strain

1-2-2 Whole cell proteomic analysi**s** of ∆*deaD* strain upon cold shock

1-2-3 SWATH mass spectrometry analysis of pre-50S/50S fractions

1-2-4 Quantitative rRNA modification analysis of pre-50S/50S fractions

**2 Workflow of Iterative classification with ab initio reconstruction in CryoSPARC**

2-1 Example in ∆*deaD* workflow

2-2 Job history in CryoSPARC

2-3 Metadata table for refined maps

**3 Parameters in PCA-UMAP and method comparison**

3-1 Direct UMAP and different metrics

3-2 PCA-UMAP with different number of principle components

3-3 Nearest neighbor in UMAP

**4 Detailed ∆*deaD* block description**

4-1 Assembly core

4-2 bL20 blocks

4-3 other blocks

**5 Dependency analysis**

5-1 Thresholding and Outlier curation

**6 rRNA modification**

**Supplementary Reference**

**Supplementary Fig. 1 | Sucrose gradient profiles showing distributions of ribosomal particles.**

10-40% Sucrose Gradient profile for cell lysates of WT (grey) and ∆*deaD* (black) grown at 19 ˚C. The intermediate peak is shown in the grey transparent box.

**Supplementary Fig. 2 | Proteomic analysis**

a) Quantitative mass spectrometry of large subunit ribosomal protein under various condition, intensity normalized to WT 37˚C. b) Volcano plot of wild type cells at 37 ˚C in M9 medium versus 19 ˚C in LB medium. Protein IDs above 1% FDR are colored in red.

**Supplementary Fig. 3 | SWATH analysis of r-protein composition in gradient fractions**

SWATH quantification of large subunit ribosomal proteins in 40S fractions (orange) and 50S fractions (black) in the Δ*deaD* strain. The error bars in this figure are generated from the standard deviation of the observed set of peptides for individual proteins (1 replicate).

**Supplementary Fig. 4 | Quantification of rRNA modification for ∆*deaD* intermediate fractions**

Relative rRNA modification level was quantified by RNA mass spectrometry and displayed as bar graph, grouped by residues and the RNase used for digestion. The bar plot includes two 40S, one 50S and one 70S fraction from the ∆*deaD* dataset.

**
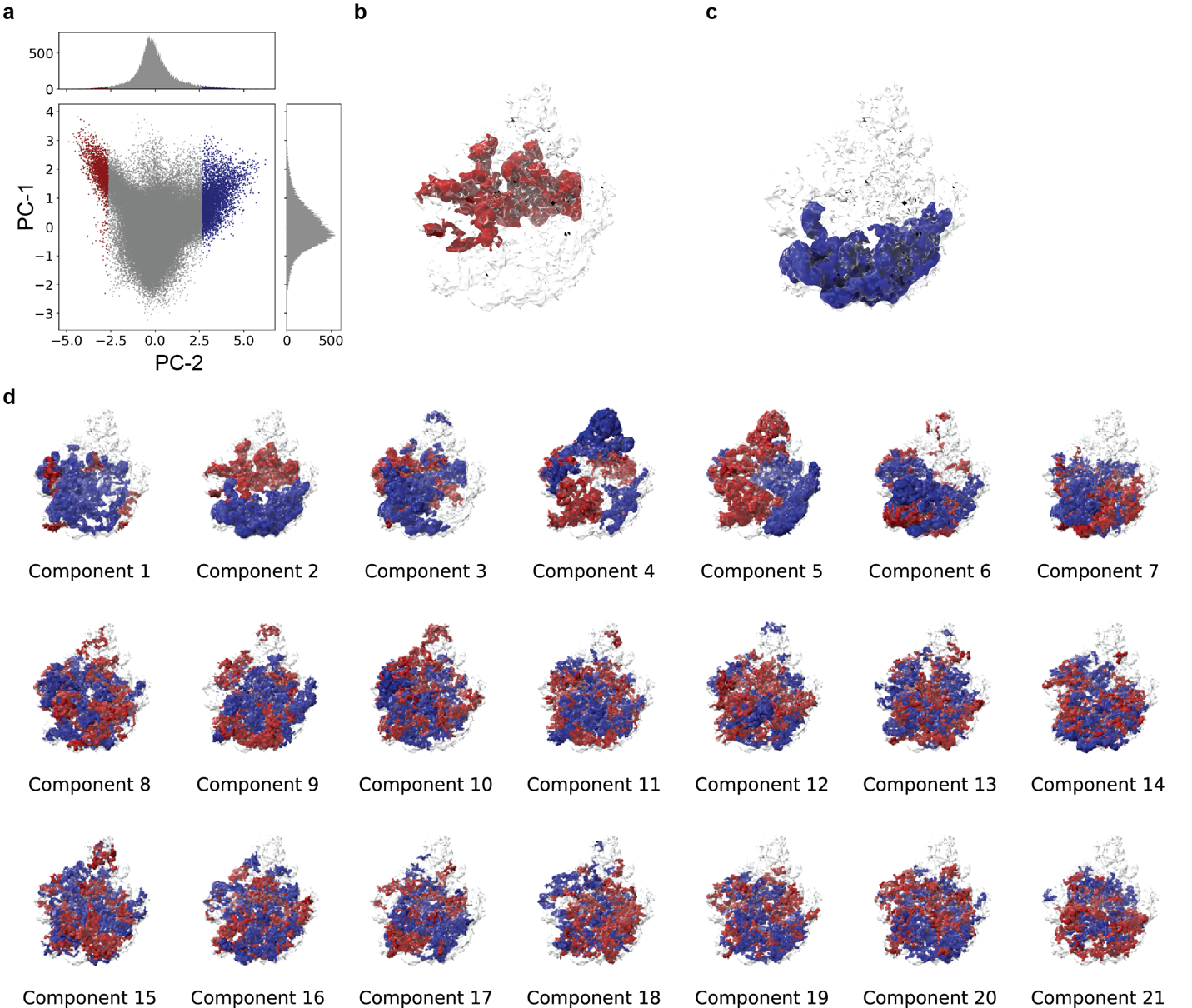
**

**Supplementary Fig. 5 | Principal Component Analysis (PCA) of voxels above threshold in the ∆*deaD* dataset** **a)** Scatterplot of the transformed value of each voxel in PC-1/PC-2 coordinates, with histograms of the marginal distributions at top and right. The extraction process is illustrated with the second principal component, PC-2. Voxels were thresholded on the PC-2 axis after thresholding at +/- 1s, resulting in volumes for a negative feature **b)** and a positive feature **c). d**) Features extracted from PC-1 to PC-21 using the same criteria in **a**). All volumes in 3D space are overlaid on the 50S subunit 1% threshold mask calculated from PDB 4ybb (transparent white).


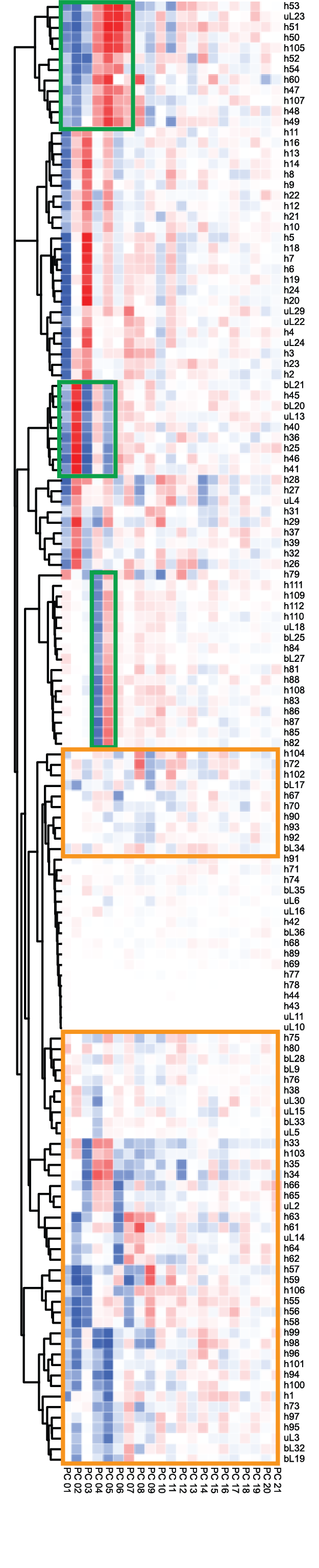


**Supplementary Fig. 6 | Occupancy matrix of positive and negative features extracted in Supplementary Fig. 5**

Voxel occupancy of PCA extracted features in 139 segments from 50S r-proteins and helices are calculated, with positive values in blue and negative values in red, and displayed in a clustered heat map. The x-axis is ordered according to the PCs, and the y-axis was ordered by hierarchical clustering. The challenges for feature extraction are highlighted by green boxes, where segments are extracted multiple times in different PCs as both negative and positive features. Orange boxes highlight cases where it is difficult to identify segments, which is particularly problematic for the higher PCs. In general, the higher PCs are primarily noise and the lower PCs are linear combinations of the desired features, but there is no straightforward way to extract the features from the PCs.


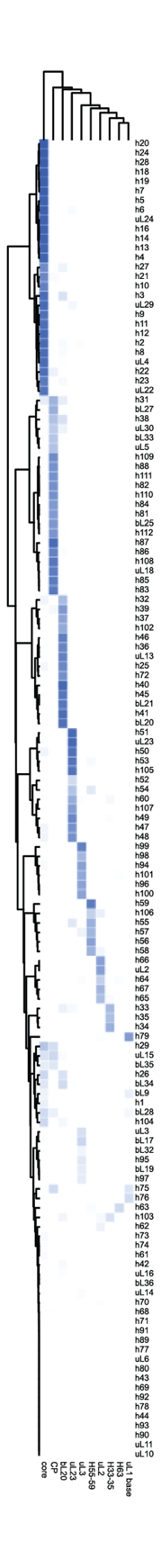


**Supplementary Fig. 7 | Occupancy matrix of blocks resulting from PCA-UMAP-HDBSCAN with in ∆*deaD* dataset**

Voxel occupancy of PCA-UMAP-HDBSCAN extracted blocks in segments from 50S r-proteins and helices are calculated and displayed in a clustered heat map. The x-and y-axes ordered by clustering, using Euclidean distance with Ward linkage. Extraction of features from the PCA-UMAP-HDBSCAN representation is straightforward.


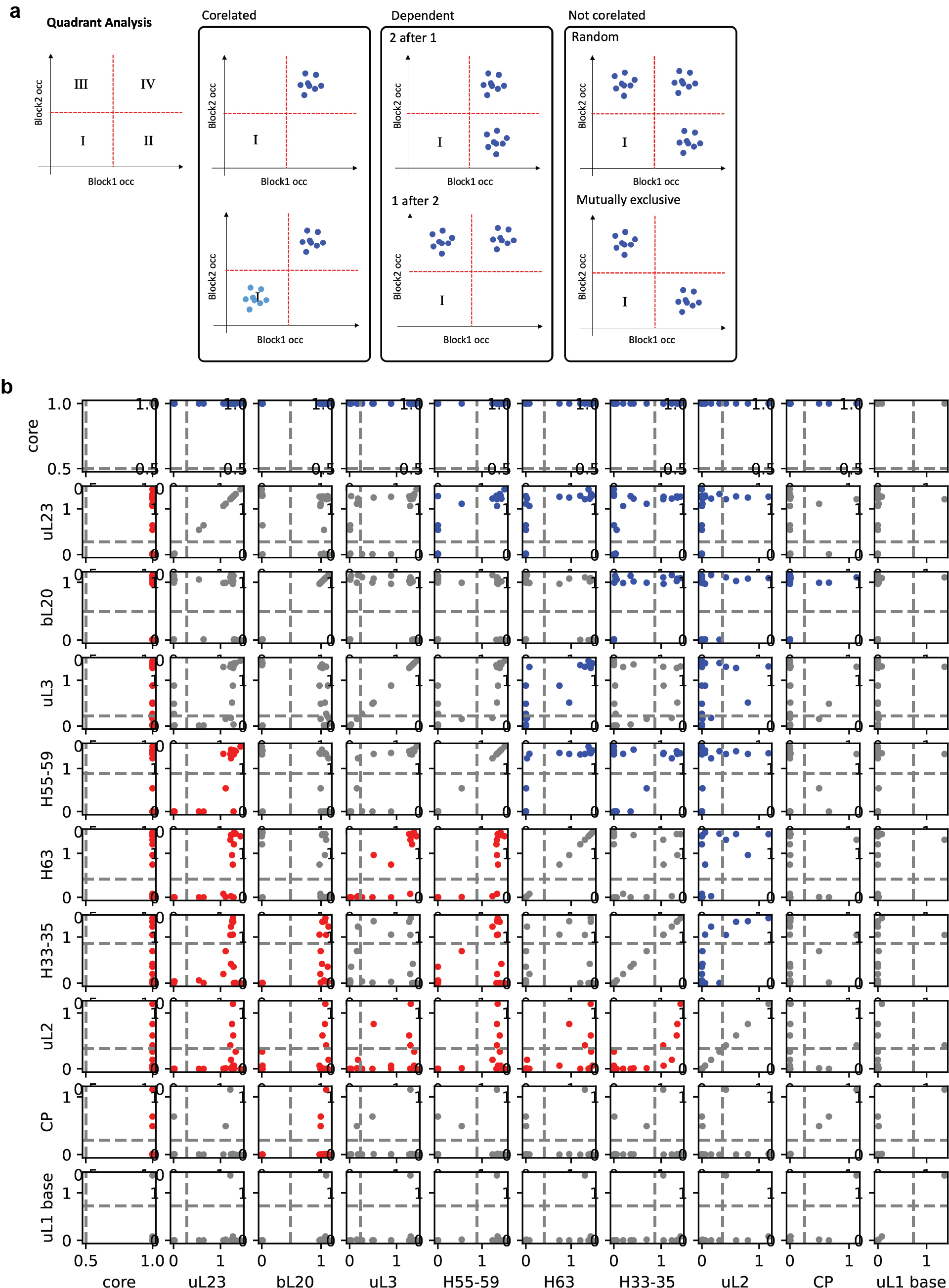


**Supplementary Fig. 8 | Quadrant analysis of assembly blocks of ∆*deaD* dataset**

**a**) Description of block dependency relationships with a quadrant plot. The scatter plot of pairwise block occupancy is divided into four quadrants (Q-I, II, III and IV). Each point represents a density map. For example, points in Q-II represent an intermediate map where block 1 is folded (occupied) while block 2 not folded (not occupied). The plots fall into three major categories, which are correlated, dependent, and not correlated. **b**) The pairwise quadrant analysis of 10 assembly blocks from ∆*deaD* dataset is displayed. Plots showing a dependency of block 1 on block 2 are colored in red, and plots showing a dependency of block 2 on block 1 are shown in blue, while uncorrelated plots are shown in gray. (See Methods for details)


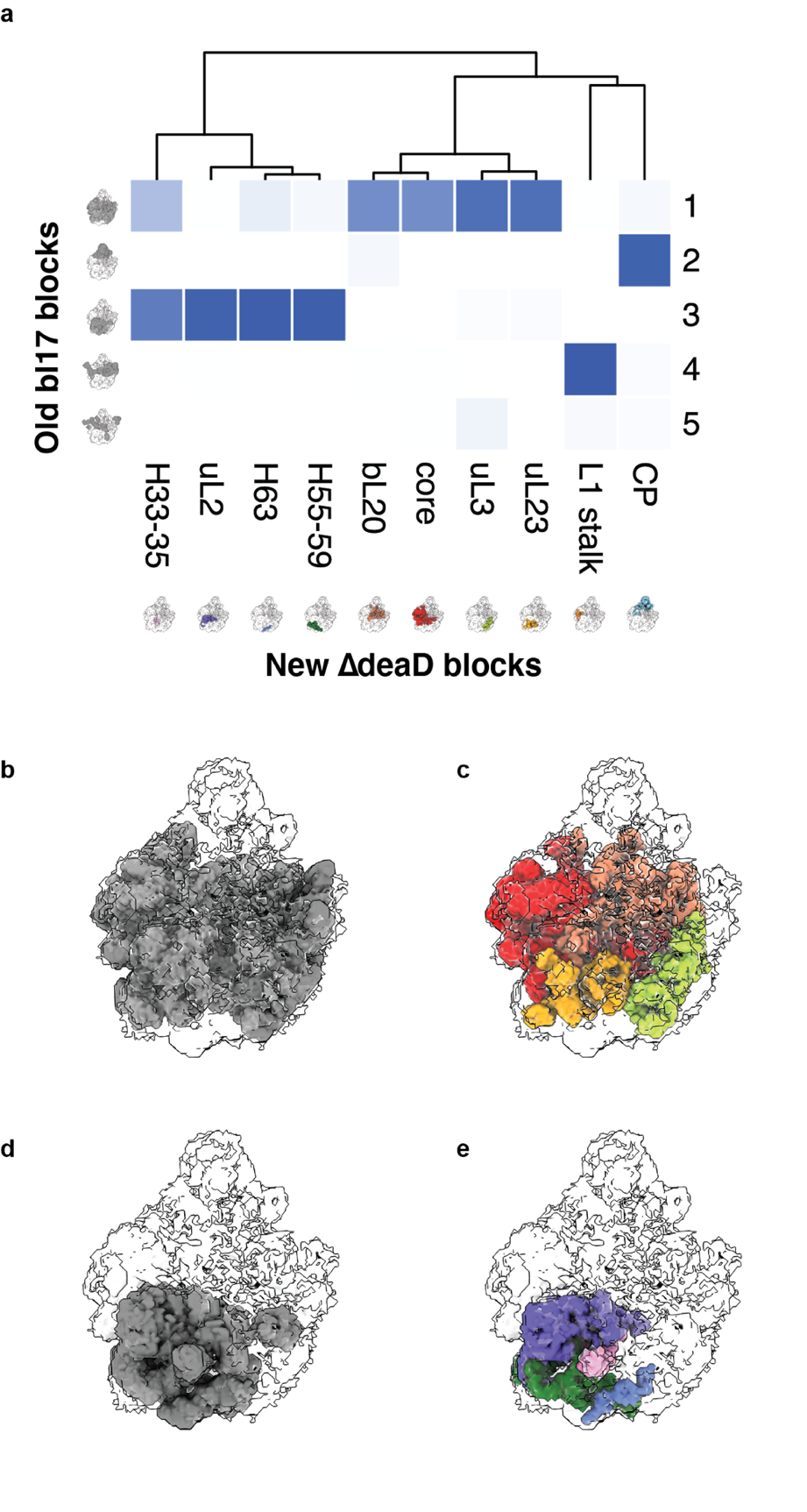


**Supplementary Fig. 9 | Subdivision of Previous bL17 blocks**  **a**) Voxel occupancy map of new blocks in previous bL17 blocks. Display of the 3D volumes shows that the previous bL17 old block 1 (**b**) can be subdivided into the assembly core, bL20, uL23 and uL3 blocks (**c**). Similarly, the previous bL17 block 3 (**d**) can be subdivided into the H55-58, H33-35, H63 and uL2 blocks (**e**).


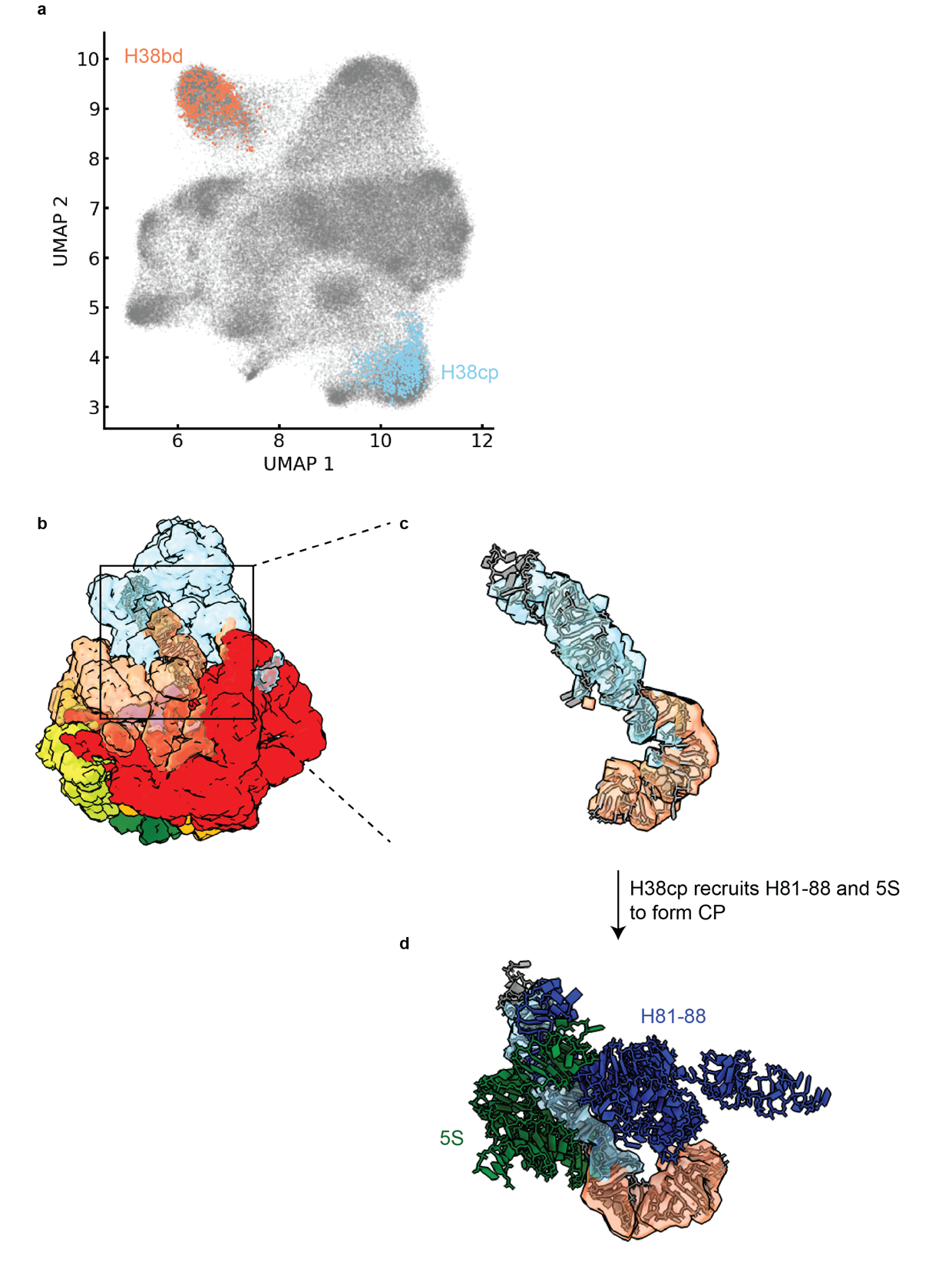


**Supplementary Fig. 10 | Subdivision of H38 led to insights into CP formation**

**a**) Scatter plot of UMAP1/2 with H38bd and H38cp colored according to their respective blocks, showing clean separation of voxels. **b**) The atomic model of H38 from 4YBB (grey) was aligned to the assembly blocks (colored according to Fig. 2), resulted in subdivision of H38 into a body part (H38bd) as part of the bL20 block and CP part (H38cp) as part of the CP block (**c**). (**d**) The atomic model of 5S (green) and H81-88 (dark blue) are displayed, showing close contact with H38cp.


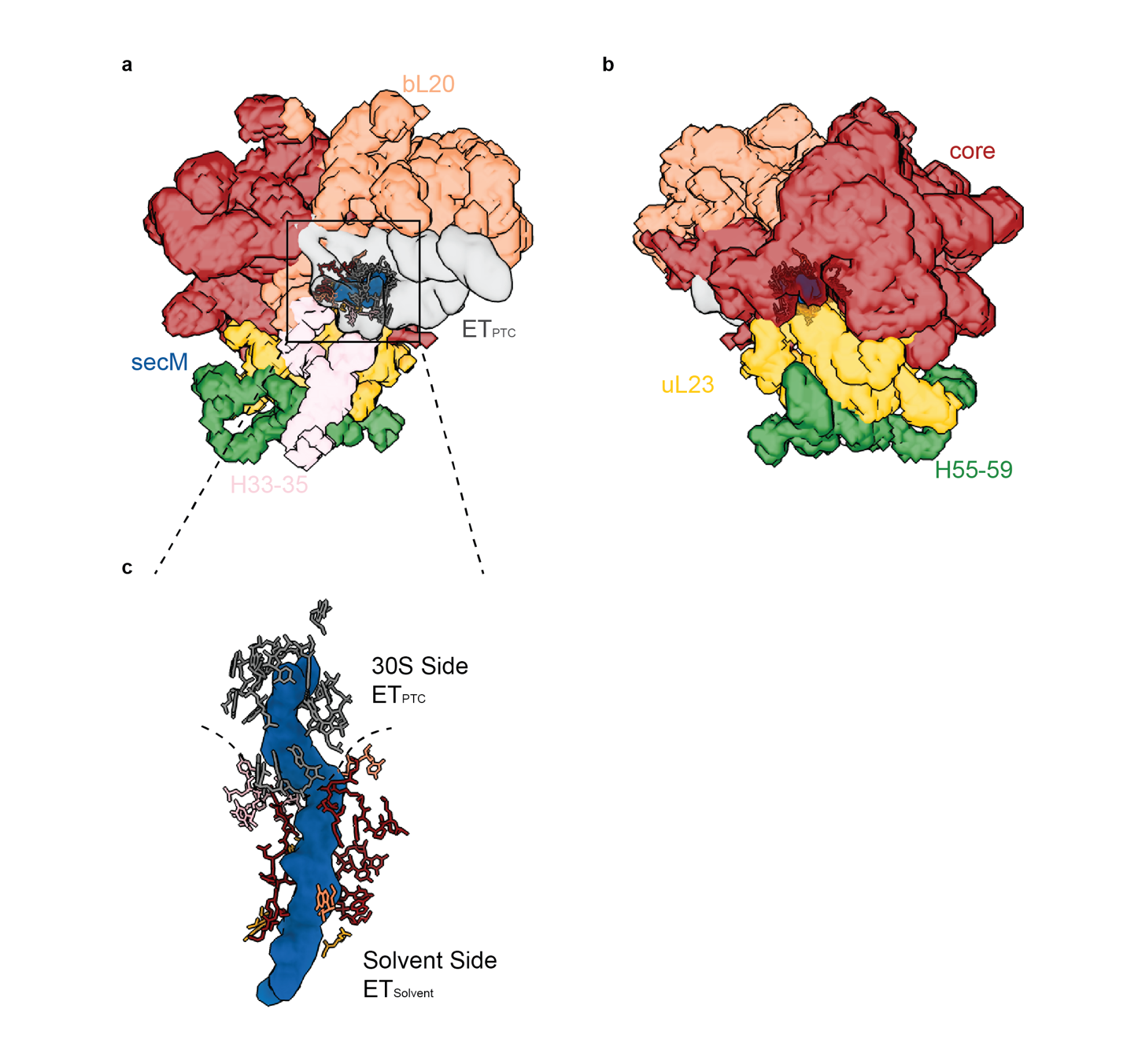


**Supplementary Fig. 11 | ET_Solvent_ and ET_PTC_**

**a-b**) Blocks surrounding the peptide exit tunnel are shown, with direct contact residues in stick form. The ETsolvent blocks are colored according to Fig. 2, the ET_PTC_ is colored in grey, and the SecM nascent chain density (molmap from PDB: 3JBU) is colored in dark blue. **c**) Contacting residues are displayed around the SecM nascent chain using same color coding in **a-b**).


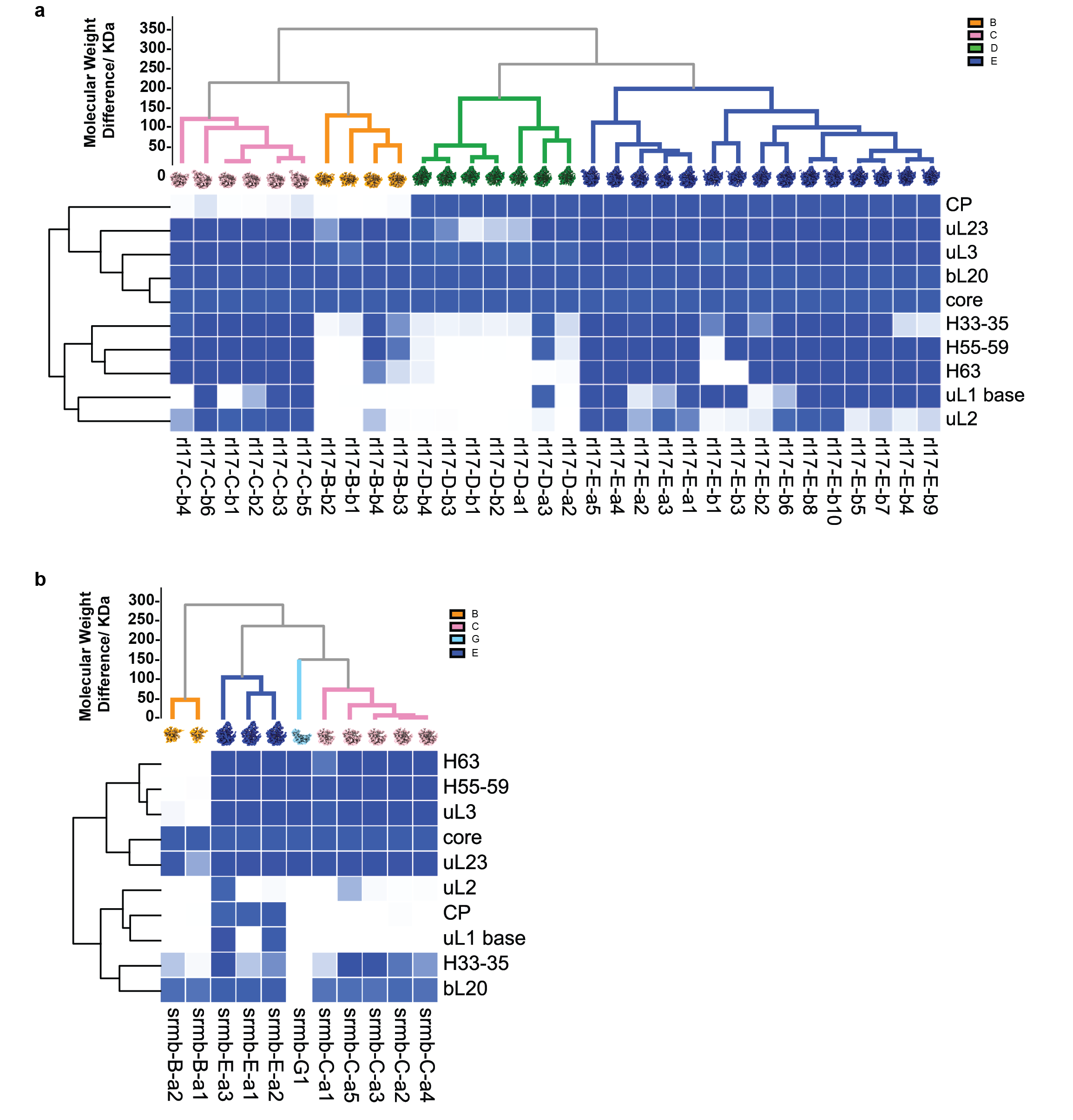


**Supplementary Fig. 12 | Comparison of intermediates from ∆*srmB* and bL17 depletion with blocks from the ∆*deaD* dataset.** The density maps from previous studies were analyzed as an occupancy matrix based on the assembly blocks from the ∆*deaD* dataset, clustering the matrix along both axes. The dendrograms are colored according to the major classes, B,C,D, E, and G. (**a**) bL17-depletion (**b**) ∆*srmB* datasets.

**
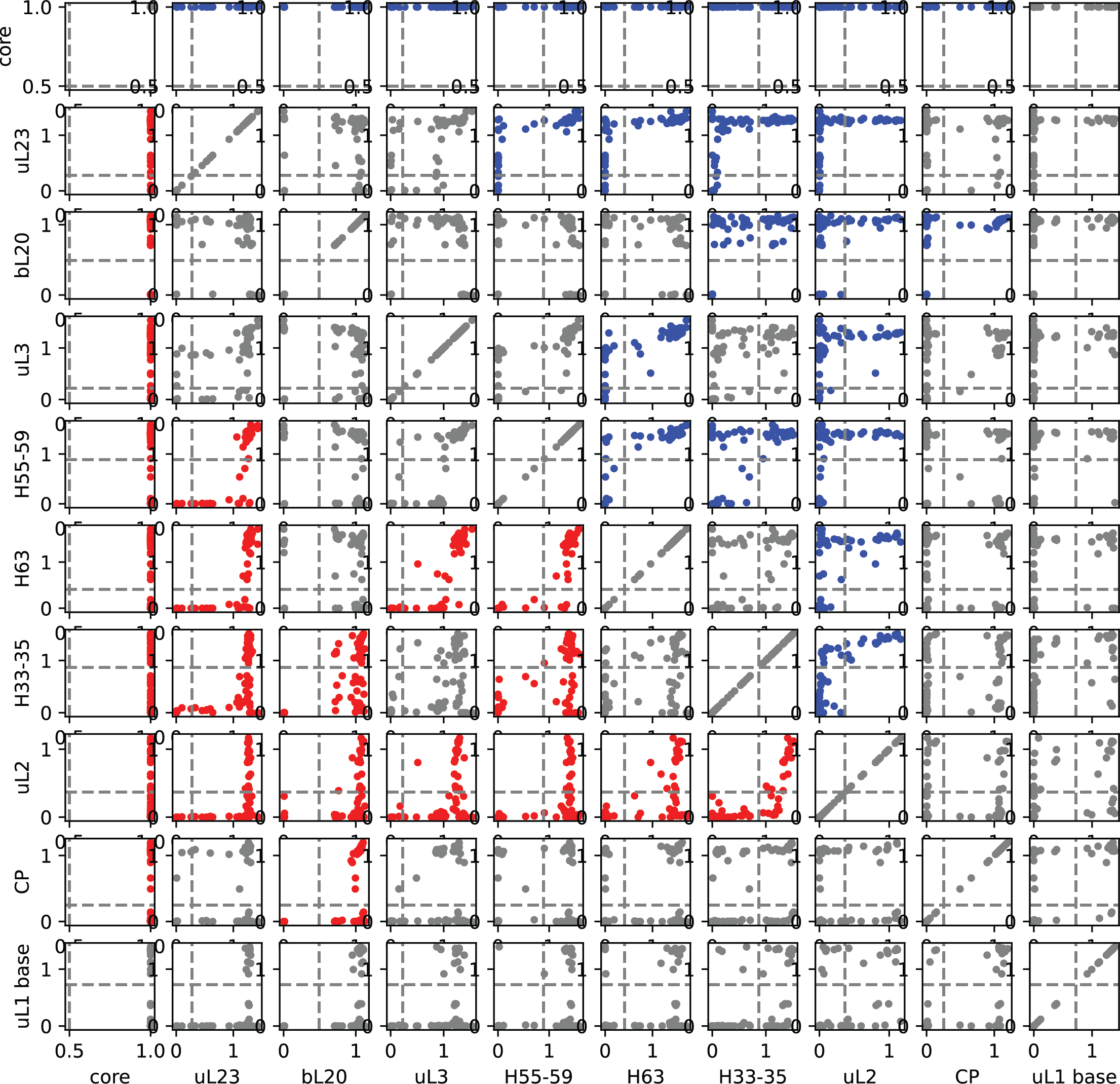
**

**Supplementary Fig. 13 | Quadrant analysis to determine the dependency of assembly blocks for three datasets.** Pairwise quadrant analysis of the 10 assembly blocks from the ∆*deaD* dataset on three datasets (64 maps) is displayed. Color coding is same as Supplementary Fig. 4.

**
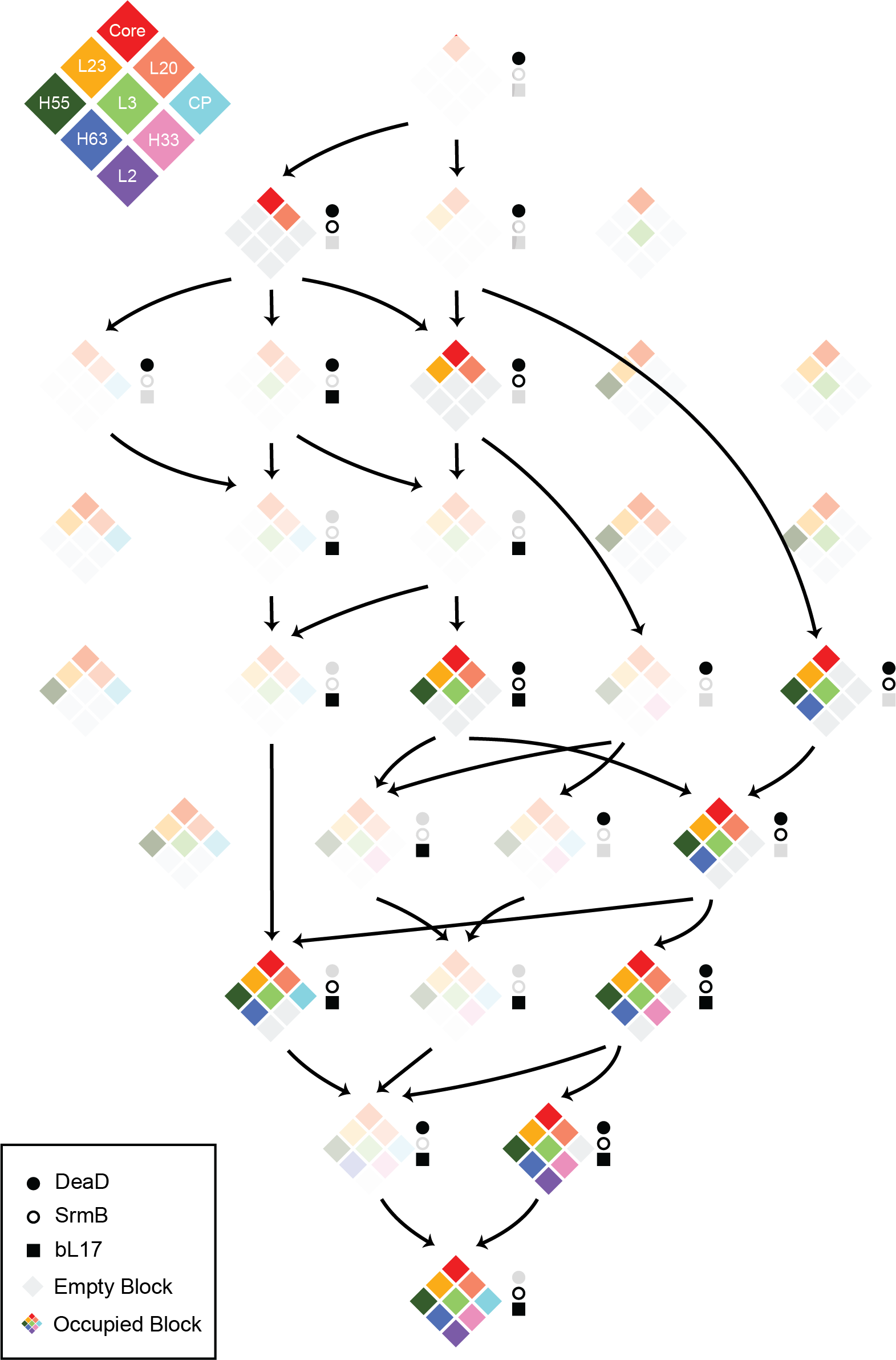
**

**Supplementary Fig. 14 | Diamond block assembly diagram for the** **∆*srmB* intermediates.**

All intermediate found in ∆*srmB* are highlighted, with color coding the same as in Fig. 4.

**
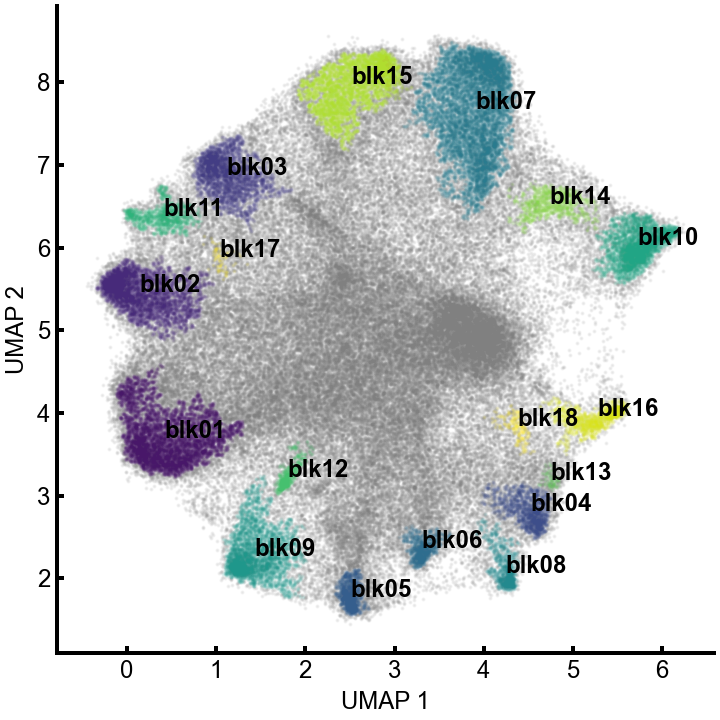
**

**Supplementary Fig. 15 | 18 blocks PCA-UMAP-HDBSCAN.** The PCA-UMAP-HDBSCAN segmentation was calculated using all three datasets, ∆*deaD*, bL17, and, ∆*srmB*. Voxels above a 1% threshold in three datasets are well organized in the newly calculated UMAP space, with 18 contiguous volume blocks extracted by clustering with HDBSCAN colored. Extraction of features from the PCA-UMAP-HDBSCAN representation is straightforward.


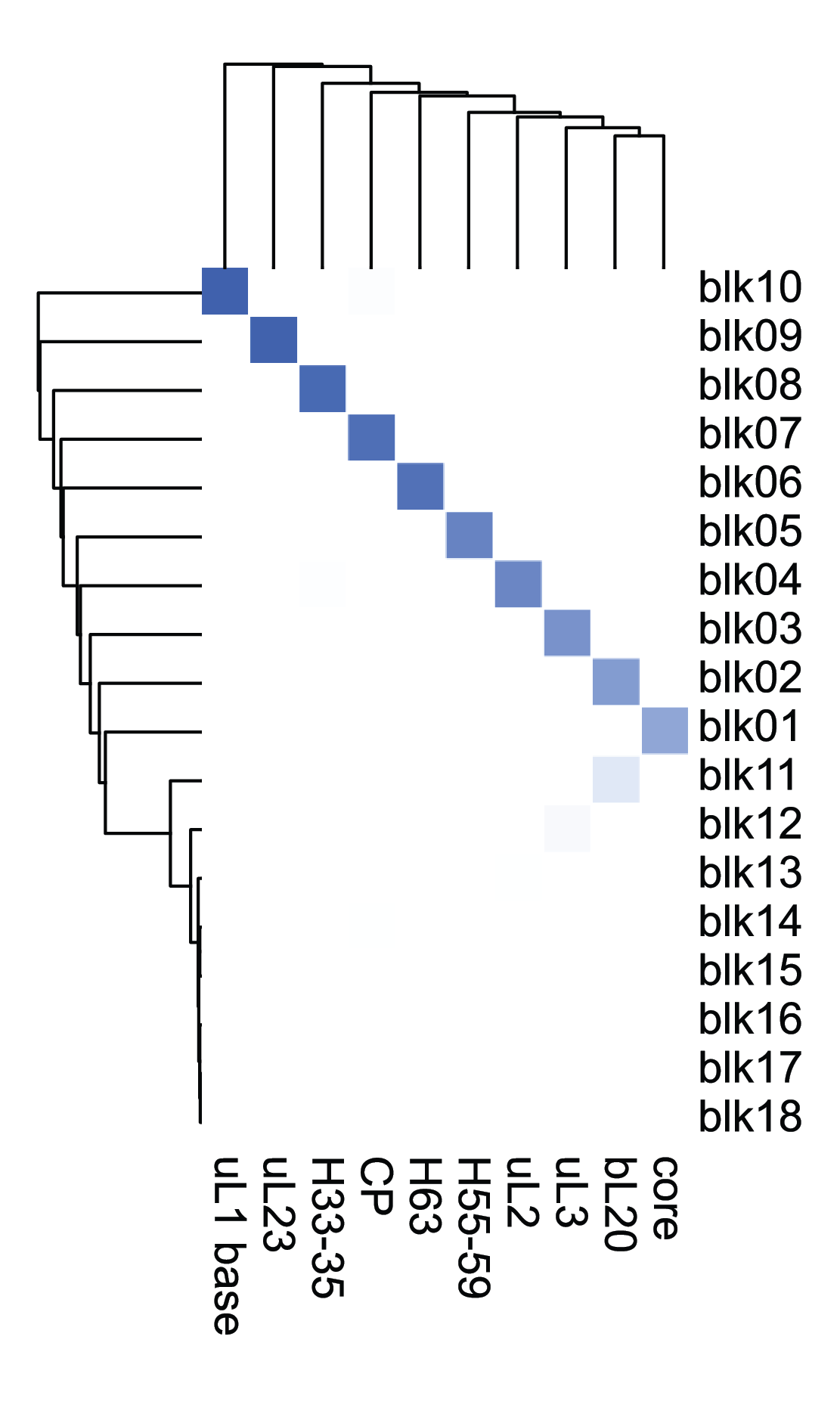


**Supplementary Fig. 16 | Occupancy analysis of 18 assembly blocks derived from all three datasets.**  Voxel occupancy matrix for the new segmentation with 18 blocks in terms of the 10 blocks from ∆*deaD* dataset, and displayed with clustering of both dimensions using Euclidean distance with Ward linkage. The extracted features are very similar, with some subdivision of the smaller set of blocks.


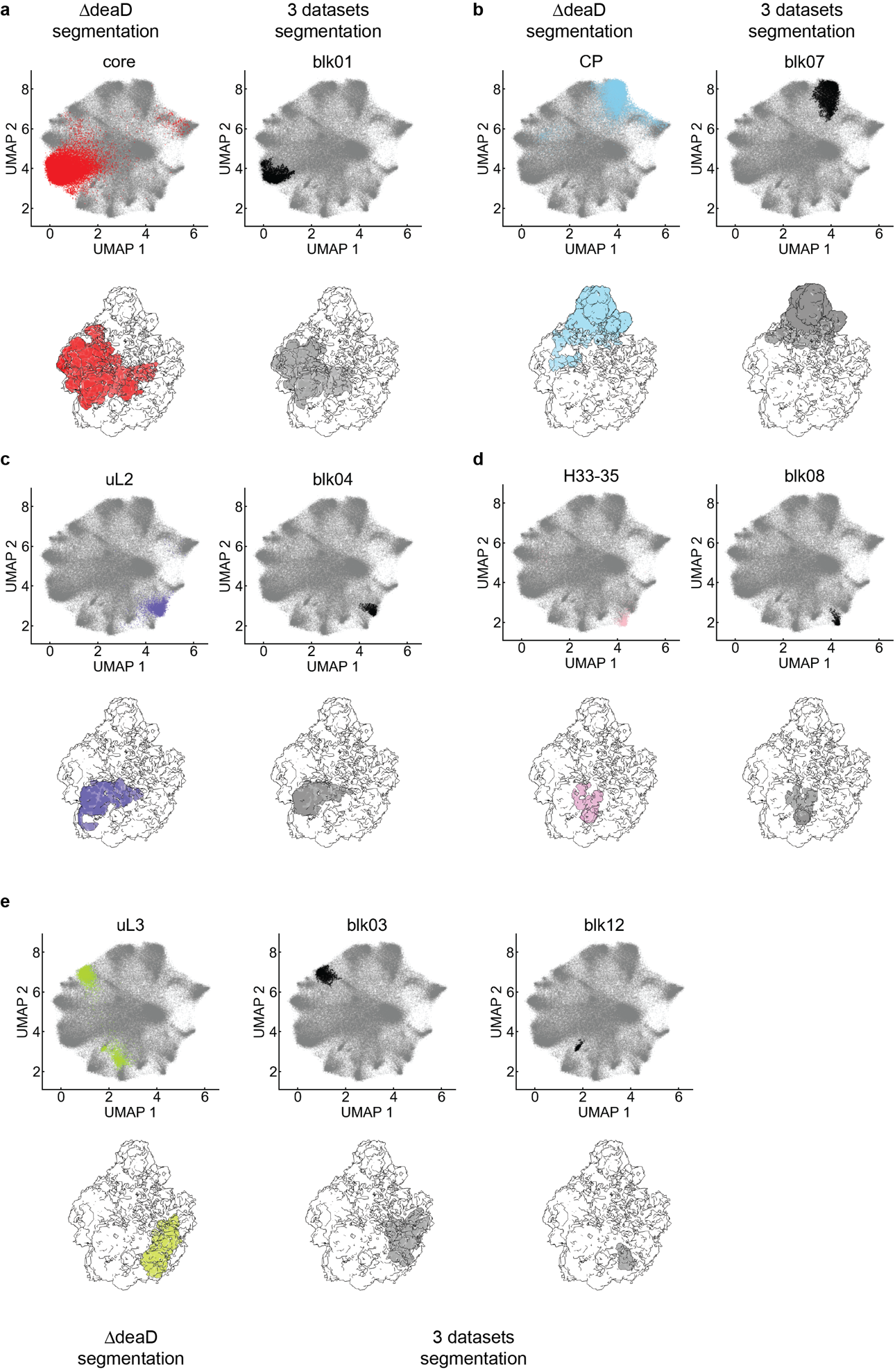


**
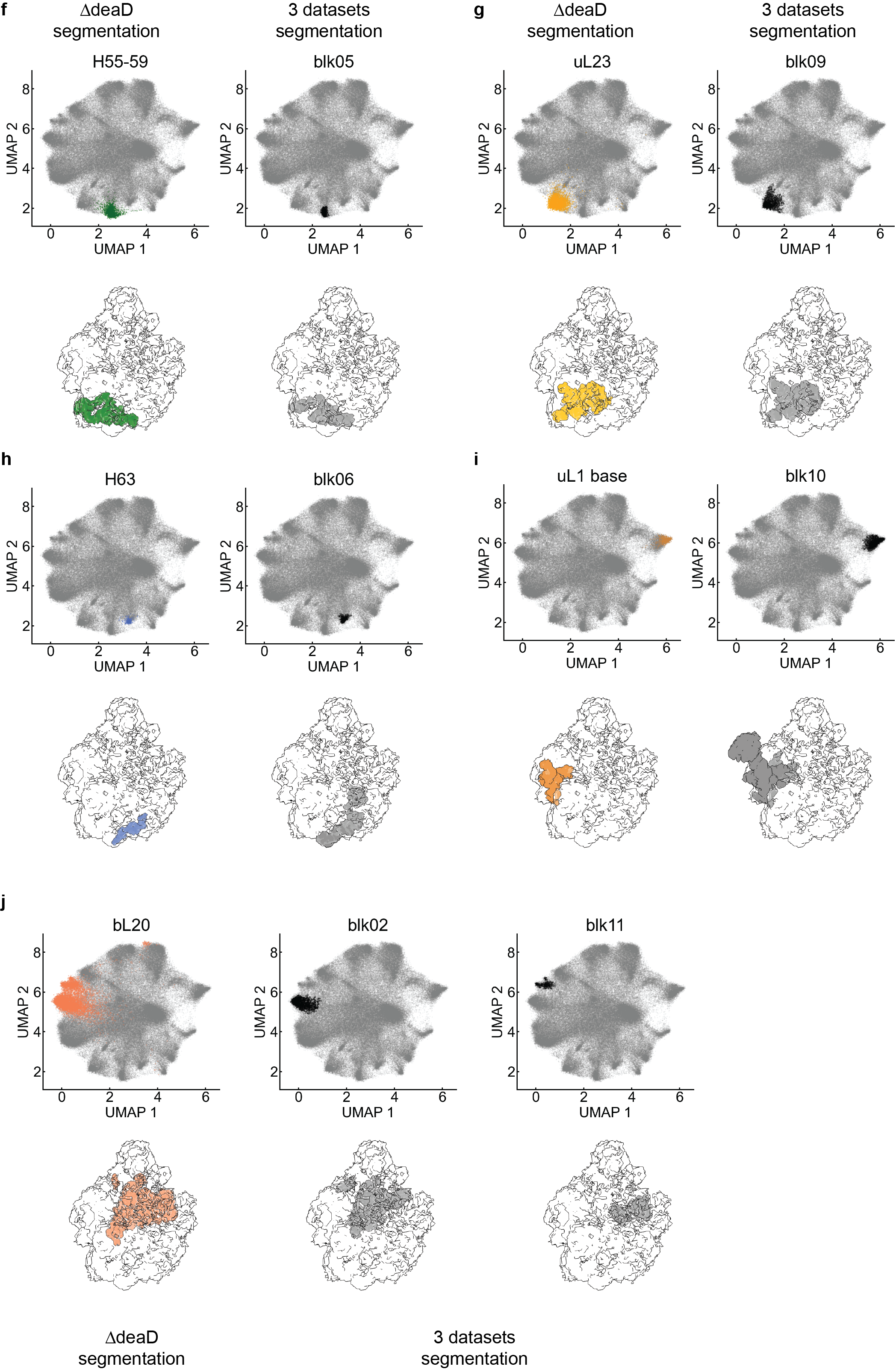
**

**Supplementary Fig. 17 | Comparison of 18 blocks from PCA-UMAP-HDBSCAN analysis of three datasets with 10 blocks from the** ∆***deaD* dataset.** Among the 18 blocks obtained from three datasets, 12 blocks are corresponding to 10 blocks from the ∆*deaD* dataset alone. For each comparison (a-d, f-i), on the top left, ∆*deaD* blocks are colored according to Fig. 2, while corresponding three dataset blocks are on the top right colored in black in UMAP1/2 scatterplots. the corresponding 3D volumes outlined in the 99-percentile threshold 50S, with ∆*deaD* blocks colored according to Fig.2 and the new 18 blocks colored in grey. Notably, for e and j, two cases of subdivision of a ∆*deaD* block are displayed with the same color coding as above.


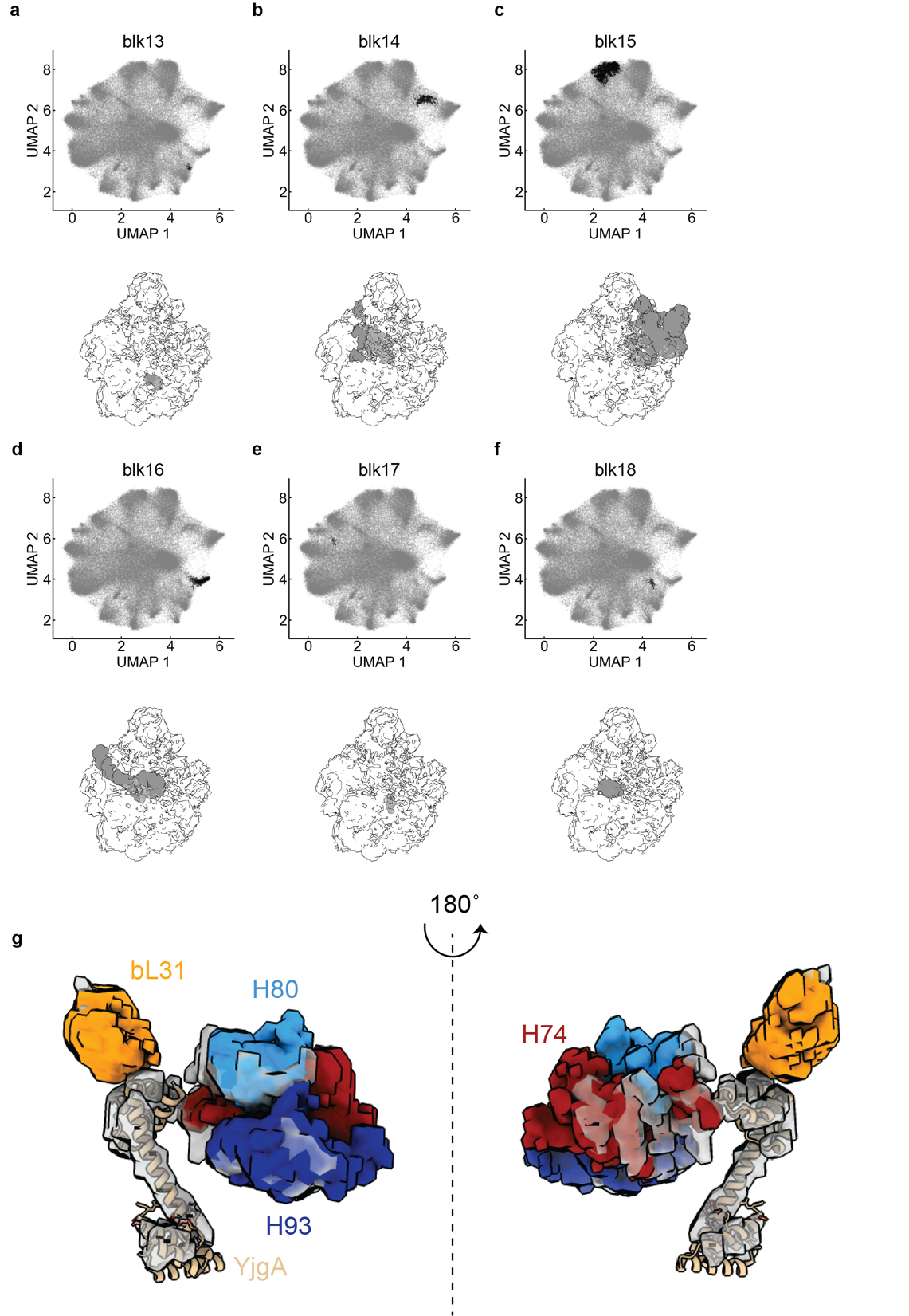


**Supplementary Fig. 18 | Six new blocks identified from the PCA-UMAP-HDBSCAN analysis of three datasets.**

**a-f**) display six new blocks in three dataset PCA-UMAP-HDBSCAN, both in UMAP space and as 3D volumes. The composition of the volumes in terms of 50S helices and proteins are described in the SI. g) Display of the volume corresponding to non-native density, identified as YjgA in blk14 (transparent grey), aligned to the atomic model of YjgA in yellow. Density for bL31, H74, H80 and H93 volumes from 4YBB are also shown in solid colored volume.

**Supplementary Fig. 19 | Examples of subclassification in analysis of ∆*deaD* intermediates.**

**a-d)** Three perpendicular cross sections of the four initial classes of ab initio reconstruction (Job J62 in **Supplementary Fig. 20**). 2D classification for particles resulting from Job J62 from **e)** class 0, **f)** class 1 , **g)** class 2, and **h)** class 3.

**Supplementary Fig. 20| Screenshot of CryoSPARC workflow for the ∆*deaD* dataset analysis.**

A case of termination of subdivision using *ab initio* reconstruction highlighted with a green rectangle. An example of merging two similar classes that emerged in highlighted by a red rectangle. The final stage of 3D refinement and map validation by ThreeDFSC is highlighted by a purple rectangle.

**Supplementary Fig. 21 | Comparison of direct UMAP and PCA-UMAP analysis using different metrics.** The ∆*deaD* dataset was subjected to direct UMAP (**a-d**) or PCA-UMAP (**e-h**) of the set of voxels above threshold, with different metrics in UMAP (**a,e** for Canberra, **b,f** for Euclidean, **c,g** for cosine and **d,h** for correlation metric). For each approach, all voxels are colored in grey in the upper panel, and are colored according to the blocks assigned Fig.2 in the lower panel in corresponding UMAP1/2 space, for comparison.

**Supplementary Fig. 22 | Boxplot of distance to block median for the 10 blocks from different approaches**

Each boxplot displays the distribution of mean distance to the block median, for each of of 10 blocks, labeled as p- for PCA-UMAP, d- for direct UMAP. can for Canberra, euc for Euclidean, cos for cosine, and cor for correlation. A box is drawn from the first quartile (Q1) to the third quartile (Q3), with a line indicating the median (Q2) inside the box. The whiskers are the max/min of the range (single replicate).

**Supplementary Fig. 23| PCA-UMAP analysis with different numbers of PCs**

Different numbers of (from 2 to 21) PCs from ∆*deaD* dataset were input into UMAP analysis using the Euclidean metric. In panel **a** all voxels as shown in gray, and in panel **b**, the voxels are colored according to Figure 2 for the 10 blocks as a reference.

**Supplementary Fig. 24| PCA-UMAP analysis with different numbers of PCs**

Different numbers of (from 2 to 21) PCs from ∆*deaD* dataset were input into UMAP analysis using the Canberra metric. In panel **a** all voxels as shown in gray, and in panel **b**, the voxels are colored according to Figure 2 for the 10 blocks as a reference.


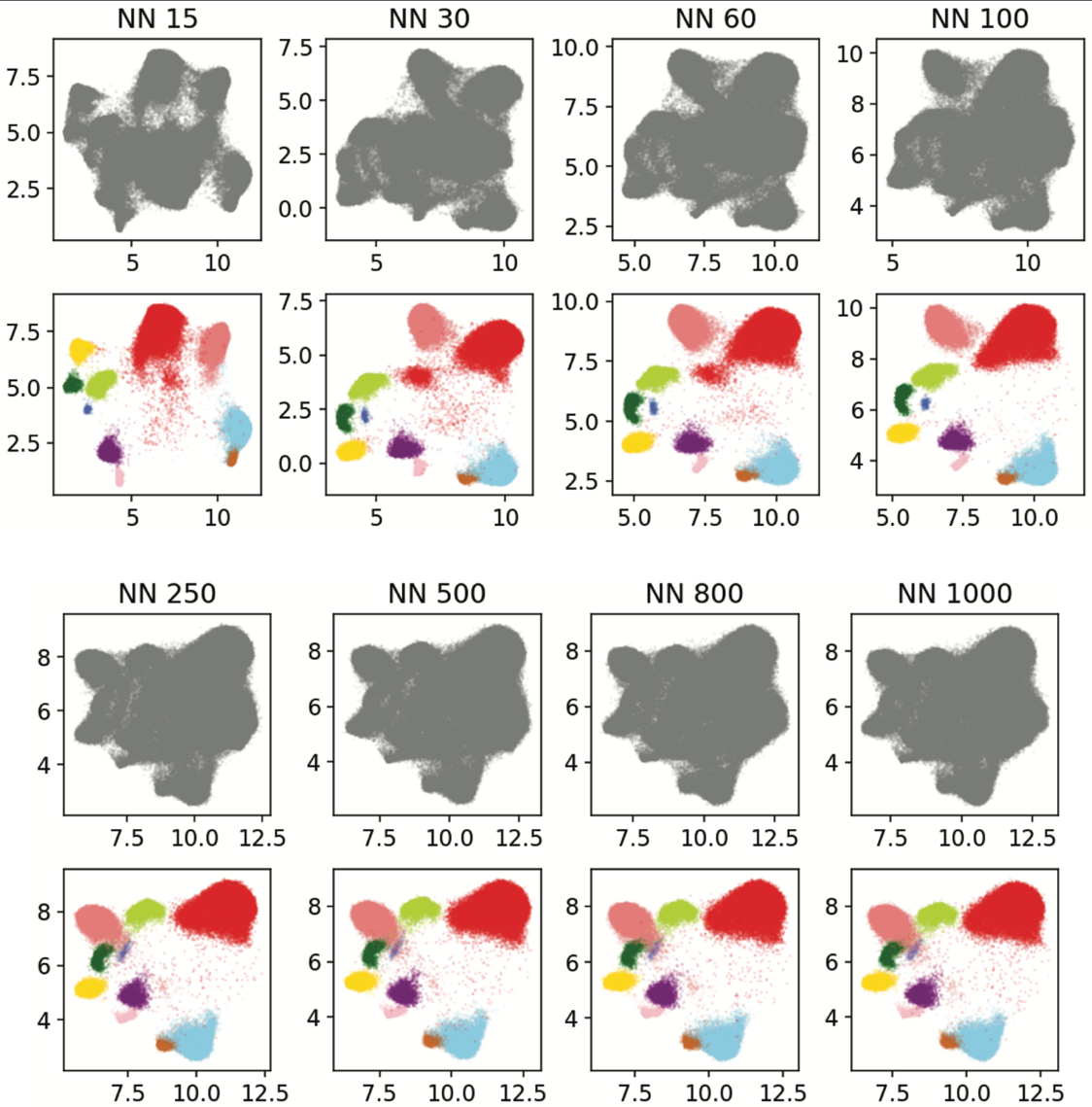


**Supplementary Fig. 25 | PCA-UMAP analysis with different numbers of nearest neighbors**

PCA-UMAP analyisis of the ∆*deaD* dataset using the Canberra metric, with different numbers of nearest neighbors for the UMAP (15, 30, 60, 100, 250, 500, 800 and 1000).

In the upper panel all voxels as shown in gray, and in the lower panel, the voxels are colored according to Figure 2 for the 10 blocks as a reference.

**Supplementary Fig. 26 | Stability test of the UMAP and PCA-UMAP analysis. a**) Mean square displacement of each voxel in 100 independent trials of the direct UMAP (x-axis) and PCA-UMAP (y-axis) calculations, show as a scatterplot with histograms of the marginal distributions at top and right. Representative plots of four consecutive calculations with direct UMAP (**b**) and PCA-UMAP (**c**). The black triangle points out the reorganization of the CP and uL1 blocks in different runs with the direct UMAP method.


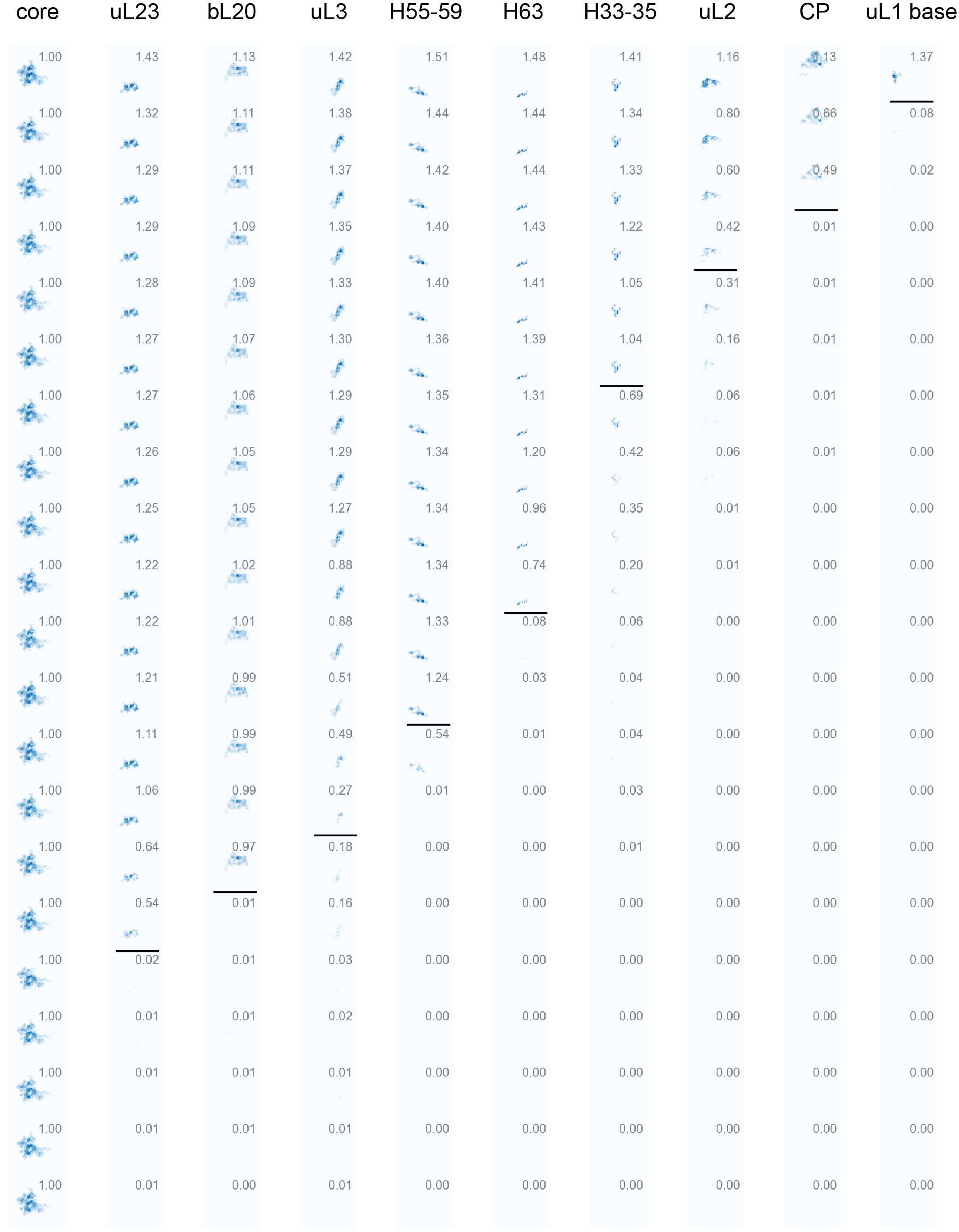


**Supplementary Fig. 27 | Thresholding the presence of 10 blocks in the set of 21 ∆*deaD* density maps** Each column shows a heatmap of the set of 21 ∆*deaD* maps after masking by one certain block, ordered from largest occupancy to smallest normalized occupancy of the blocks displayed near the heatmap. The thresholding line is drawn in black for each block.

**Supplementary Fig. 28 | Scheme for dependency map pruning.** Dependencies that emerge from the quadrant analysis can be represented as directed graphs. To simplify the graph network, edges that short-circuit other paths are pruned, as illustrated for a simple set of 3 nodes with dependences 1->2, 1->3, 2->3.

**Supplementary Table 1** Doubling time of different strain at 19 ˚C

| **Strain** | **Plasmid** | **Medium** | **HSL conc. (nM)** | **Doubling Time (min)^*^** |
| --- | --- | --- | --- | --- |
| BW25113 | - | LB | 0 | 122 |
| Δ*deaD* | - | LB | 0 | 370 |
| Δ*deaD* | pHSL-*deaD*^*^ | LB | 0 | 163 |
| Δ*deaD* | pHSL-*deaD* | LB | 0.1 | 177 |
| Δ*deaD* | pHSL-*deaD* | LB | 0.5 | 199 |
| Δ*deaD* | pHSL-*deaD* | LB | 1 | 189 |

**^*^** pHSL-*deaD* plamid is constructed from pHSL-*rplQ*, replacing *rplQ* with *deaD*.
**^* *^** Doubling time was fitted in Prism using non-linear regression (exponential growth)

Single colony was picked from LB agar plates and inoculate in LB medium at 37 ^o^C overnight. The next day, overnight culture was diluted (1:200) into 500 mL LB. The growing curve was recorded at 19 ˚C with vigorously shaking.

**Supplementary Table 2** intermediate density map metadata

| **Job ID** | **Class** | **Subclass** | **Corrected Resolution** | **Particle Number** | **Voxel** | **In dataset name** | **Name** | **Project ID** | **EMDB ID** |
| --- | --- | --- | --- | --- | --- | --- | --- | --- | --- |
| 307 | B | B-a | 5.91 | 2490 | 22043 | B-a1 | dead-B-a1 | 11 | EMD-40517 |
| 309 | B | B-a | 5.41 | 3303 | 22847 | B-a2 | dead-B-a2 | 11 | EMD-40519 |
| 308 | B | B-a | 5.62 | 3125 | 22867 | B-a3 | dead-B-a3 | 11 | EMD-40520 |
| 311 | B | B-a | 5.41 | 8604 | 25437 | B-a4 | dead-B-a4 | 11 | EMD-40524 |
| 312 | B | B-a | 6.24 | 1911 | 28430 | B-a5 | dead-B-a5 | 11 | EMD-40526 |
| 310 | B | B-a | 5.99 | 1387 | 31601 | B-a6 | dead-B-a6 | 11 | EMD-40528 |
| 313 | B | B-b | 6.43 | 1689 | ﻿27444 | B-b1 | dead-B-b1 | 11 | EMD-40530 |
| 299 | C | C-a | 6.14 | 2028 | 31141 | C-a1 | dead-C-a1 | 11 | EMD-40532 |
| 300 | C | C-a | 5.96 | 2112 | 32584 | C-a2 | dead-C-a2 | 11 | EMD-40534 |
| 301 | C | C-a | 6.16 | 1763 | 33412 | C-a3 | dead-C-a3 | 11 | EMD-40536 |
| 302 | C | C-a | 5.41 | 6810 | 33905 | C-a4 | dead-C-a4 | 11 | EMD-40538 |
| 298 | C | C-a | 6.27 | 1500 | 36134 | C-a5 | dead-C-a5 | 11 | EMD-40540 |
| 303 | C | C-b | 5.41 | 2416 | 41387 | C-b1 | dead-C-b1 | 11 | EMD-40542 |
| 304 | C | C-b | 5.41 | 3338 | 37531 | C-b2 | dead-C-b2 | 11 | EMD-40544 |
| 293 | E | E-a | 5.43 | 2312 | 50393 | E-a1 | dead-E-a1 | 11 | EMD-40546 |
| 297 | G | G | 6.74 | 2017 | 25794 | G1 | dead-G1 | 11 | EMD-40548 |
| 296 | G | G | 5.89 | 5464 | 26579 | G2 | dead-G2 | 11 | EMD-40550 |
| 294 | G | G | 5.41 | 5758 | 29195 | G3 | dead-G3 | 11 | EMD-40552 |
| 295 | G | G | 5.74 | 3019 | 30902 | G4 | dead-G4 | 11 | EMD-40555 |
| 306 | B | preB | 6.39 | 2837 | 13990 | preB1 | dead-preB1 | 11 | EMD-40551 |
| 314 | B | preB | 7.36 | 1103 | ﻿16696 | preB2 | dead-preB2 | 11 | EMD-40549 |
| 1831 | B | B-b | 5.45 | 2685 | 30703 | B-b1 | rl17-B-b1 | 26 | EMD-40309 |
| 1830 | B | B-b | 6.00 | 1606 | 31052 | B-b2 | rl17-B-b2 | 26 | EMD-40311 |
| 1832 | B | B-b | 5.45 | 1772 | 32554 | B-b3 | rl17-B-b3 | 26 | EMD-40313 |
| 1833 | B | B-b | 5.45 | 1585 | 35814 | B-b4 | rl17-B-b4 | 26 | EMD-40511 |
| 1814 | C | C-b | 5.45 | 3068 | 39005 | C-b1 | rl17-C-b1 | 26 | EMD-40314 |
| 1815 | C | C-b | 5.45 | 2943 | 39801 | C-b2 | rl17-C-b2 | 26 | EMD-40315 |
| 1811 | C | C-b | 5.45 | 4368 | 41135 | C-b3 | rl17-C-b3 | 26 | EMD-40317 |
| 1813 | C | C-b | 5.54 | 1277 | 41342 | C-b4 | rl17-C-b4 | 26 | EMD-40319 |
| 1812 | C | C-b | 5.45 | 3438 | 43118 | C-b5 | rl17-C-b5 | 26 | EMD-40321 |
| 1810 | C | C-b | 5.45 | 4655 | 45238 | C-b6 | rl17-C-b6 | 26 | EMD-40323 |
| 1801 | D | D-a | 5.45 | 2084 | 37456 | D-a1 | rl17-D-a1 | 26 | EMD-40327 |
| 1802 | D | D-a | 5.45 | 2377 | 40580 | D-a2 | rl17-D-a2 | 26 | EMD-40329 |
| 1803 | D | D-a | 5.45 | 1964 | 42170 | D-a3 | rl17-D-a3 | 26 | EMD-40331 |
| 1804 | D | D-b | 5.45 | 1651 | 42152 | D-b1 | rl17-D-b1 | 26 | EMD-40333 |
| 1805 | D | D-b | 5.45 | 1914 | 42857 | D-b2 | rl17-D-b2 | 26 | EMD-40512 |
| 1806 | D | D-b | 5.45 | 1582 | 43879 | D-b3 | rl17-D-b3 | 26 | EMD-40514 |
| 1807 | D | D-b | 5.45 | 1506 | 45093 | D-b4 | rl17-D-b4 | 26 | EMD-40516 |
| 1819 | E | E-a | 5.45 | 2468 | 47104 | E-a1 | rl17-E-a1 | 26 | EMD-40518 |
| 1821 | E | E-a | 5.45 | 1554 | 47445 | E-a2 | rl17-E-a2 | 26 | EMD-40521 |
| 1820 | E | E-a | 5.45 | 1921 | 48373 | E-a3 | rl17-E-a3 | 26 | EMD-40523 |
| 1835 | E | E-a | 5.45 | 11791 | 48927 | E-a4 | rl17-E-a4 | 26 | EMD-40525 |
| 1816 | E | E-a | 5.45 | 13258 | 52135 | E-a5 | rl17-E-a5 | 26 | EMD-40527 |
| 1809 | E | E-b | 5.45 | 6390 | 47629 | E-b1 | rl17-E-b1 | 26 | EMD-40529 |
| 1829 | E | E-b | 5.45 | 1949 | 55635 | E-b10 | rl17-E-b10 | 26 | EMD-40531 |
| 1822 | E | E-b | 5.68 | 1273 | 49021 | E-b2 | rl17-E-b2 | 26 | EMD-40533 |
| 1808 | E | E-b | 5.45 | 1532 | 50798 | E-b3 | rl17-E-b3 | 26 | EMD-40535 |
| 1825 | E | E-b | 5.45 | 1968 | 52095 | E-b4 | rl17-E-b4 | 26 | EMD-40537 |
| 1823 | E | E-b | 5.45 | 1467 | 52913 | E-b5 | rl17-E-b5 | 26 | EMD-40539 |
| 1827 | E | E-b | 5.45 | 1428 | 53397 | E-b6 | rl17-E-b6 | 26 | EMD-40541 |
| 1824 | E | E-b | 5.45 | 1892 | 53474 | E-b7 | rl17-E-b7 | 26 | EMD-40543 |
| 1828 | E | E-b | 5.45 | 1532 | 54563 | E-b8 | rl17-E-b8 | 26 | EMD-40545 |
| 1826 | E | E-b | 5.45 | 1818 | 55005 | E-b9 | rl17-E-b9 | 26 | EMD-40547 |
| 202 | B | B-a | 9.27 | 1234 | 19913 | B-a1 | srmb-B-a1 | 15 | EMD-40316 |
| 190 | B | B-a | 8.29 | 1419 | 24089 | B-a2 | srmb-B-a2 | 15 | EMD-40318 |
| 196 | C | C-a | 7.75 | 4355 | 24078 | C-a1 | srmb-C-a1 | 15 | EMD-40320 |
| 197 | C | C-a | 8.08 | 1787 | 29870 | C-a2 | srmb-C-a2 | 15 | EMD-40322 |
| 204 | C | C-a | 6.81 | 7185 | 30332 | C-a3 | srmb-C-a3 | 15 | EMD-40324 |
| 198 | C | C-a | 8.46 | 2439 | 31184 | C-a4 | srmb-C-a4 | 15 | EMD-40325 |
| 200 | C | C-a | 7.89 | 1525 | 35286 | C-a5 | srmb-C-a5 | 15 | EMD-40328 |
| 193 | E | E-a | 7.56 | 2485 | 36449 | E-a1 | srmb-E-a1 | 15 | EMD-40330 |
| 194 | E | E-a | 7.77 | 1624 | 41658 | E-a2 | srmb-E-a2 | 15 | EMD-40332 |
| 192 | E | E-a | 6.99 | 1827 | 43453 | E-a3 | srmb-E-a3 | 15 | EMD-40513 |
| 195 | G | G | 6.84 | 9918 | 24535 | G1 | srmb-G1 | 15 | EMD-40515 |

* Job or Project ID refer to CryoSPARC job or project ID.

* Classes were assigned according to Figure 1d.

* “Corrected resolution” was obtained from ThreeDFSC in Å.

* “Voxel” is voxel number above 1.00 threshold in the density map.

**Supplementary Table 3** 5 Å residues around nascent peptide chain in 3JBU and 5NWY

| **PDB** | **Entity** | **Site** | **contents** | **secondary structure** | **block** |
| --- | --- | --- | --- | --- | --- |
| 3JBU | 23S | 460-461 | AC | H23 | core |
|  |  | 470 | A | H23 |  |
|  |  | 507-508 | AA | H24 |  |
|  |  | 752 | A | H35 | H33-35 |
|  |  | 790 | U | H32 | bL20 |
|  |  | 789 | A | H103 |  |
|  |  | 1321 | A | H50 | uL23 |
|  |  | 1614 | A | H107 |  |
|  |  | 2061-2063 | GAC | H74 | PTC |
|  |  | 2451-2452 | AC | H74/H89 |  |
|  |  | 2505-2506 | GU | H90 |  |
|  |  | 2585 | U | H90 |  |
|  |  | 2609-2611 | UCC | H93/H73 |  |
|  | uL4 | 61 | R |  | core |
|  | uL22 | 82-85 | MKRI |  |  |
|  |  | 90-95 | KGRADA |  |  |
|  | uL23 | 71-72 | GQ |  | uL23 |
| 5NWY | 23S | 460-463 | ACCG | H23 | core |
|  |  | 470-471 | AA | H23 |  |
|  |  | 746-752 | UUG--AA | H35 | H33-35 |
|  |  | 790-791 | UC | H32 | bL20 |
|  |  | 1259 | G | H26 |  |
|  |  | 1323 | C | H50 | uL23 |
|  |  | 1614 | A | H107 |  |
|  |  | 1782 | U | H65 | uL2 |
|  |  | 2057-2059 | GAA | H73/74 | PTC |
|  |  | 2061-2063 | GAC | H74 |  |
|  |  | 2451 | A | H74/H89 |  |
|  |  | 2505-2506 | GU | H90 |  |
|  |  | 2581-2586 | G-GUUU | H90 |  |
|  |  | 2602 | A | H93 |  |
|  |  | 2609-2612 | UCCC | H93/H73 |  |
|  | uL4 | 57-67 | KKPWR-KGTGR | core | uL4 |
|  | uL22 | 83-95 | KRI----KGRADR | core | uL22 |

**Supplementary Table 4** Detailed descriptions of three dataset blocks

| **18 blocks** | **contents** |
| --- | --- |
| blk01 | Core block |
| blk02 | bl20 block – H25 uL13 |
| blk03 | uL3 block |
| blk04 | uL2 block |
| blk05 | H55-59 block |
| blk06 | H63 block |
| blk07 | CP block |
| blk08 | H34 |
| blk09 | uL23 |
| blk10 | uL1 base |
| blk11 | uL13 H25 |
| blk12 | bL17 |
| blk13 | H62 |
| blk14 | H74 93 80 bL33 yjgA |
| blk15 | uL10/11 stalk |
| blk16 | H68-70 |
| blk17 | H104 |
| blk18 | uL14 |

**Supplementary Table 5** Summary table for PCA-UMAP

| **Method** | **Direct PCA** | **Direct UMAP** | **PCA-UMAP(Euclidean)** | **PCA-UMAP(Canberra)** |
| --- | --- | --- | --- | --- |
| Cluster identification | No | Yes | Yes | Yes |
| Cluster extraction | No | No | spectra distribution | Yes |
| Reproducibility | Same every time | block jumping  (Canberra) | Not tested | Good |

**1-1 pre50S intermediate accumulated in ∆*deaD* strain upon cold shock**

DeaD is a cold shock protein in E. coli, with several annotated functions involving mRNA stability and association with the 50S ribosomal subunit ^1-3^. Indeed, whole cell proteomics shows DeaD is induced 4-fold upon cold shock in wild type BW25113 strain, while the abundance of most of the ribosomal proteins remains unchanged (Supplementary Fig. 2). The ∆*deaD* strain shows a severe growth defect at 19 C, but not at 37 C (Table S1), and the sucrose gradient of total cell lysate shows a decrease in the abundance of 70S ribosomes, an accumulation of free 30S subunits and new intermediate peaks arising between 30S and 50S (Supplementary Fig. 1), supporting the accumulation of pre-50S subunits, consistent with previous results ^1^. Both the growth defect and sucrose gradient profile of the deletion strain can be rescued after introducing a DeaD expression plasmid, which further supports that the assembly defect comes from absence of DeaD. The effect could be either a direct effect on 50S assembly, or indirect due to an effect of the DEAD-box in regulating some ribosomal protein expression level. Whole cell proteomics on the deletion strain at 19 C, clearly shows that there is no significant alteration of r-protein levels, which implies more direct impact of DeaD on ribosome biogenesis. The intermediate fraction was also analyzed by quantitative protein mass spectrometry, showing partial depletion of bL35, bL36, bL28, bL32, bL34, bL33, uL30 and uL6 proteins (Supplementary Fig. 3).

**1-2 Mass spectrometry methods**

**1-2-1 Doubling time of WT, ∆*deaD* and Δ*deaD*-pHSL-*deaD*** **strain**

WT, Δ*deaD* and Δ*deaD*-pHSL-*deaD* strains were inoculated in LB medium and grown overnight, then diluted into fresh 500 mL LB (1:200) at 19 or 37 ^o^C. Either 0 or 2.5 nM HSL was added into the Δ*deaD*-pHSL-*deaD* strain during cell culture. The OD_600_ was monitored during growth until mid-log stage. The time-coursed OD_600_ was used to fit the doubling time in the expression for exponential growth in GraphPad Prism using non-linear regression. Cells from the WT and ∆*deaD* culture harvested at OD_600_ = 0.3-0.5 by centrifugation, and prepared for sucrose gradient analysis (see Methods) and mass spectrometry analysis.

**1-2-2 Whole cell proteomic analysis**

WT cells were grown in ^15^N M9 medium at 37 ˚C as ^15^N spike for quantitation. For Δ*deaD* cells grown at 19 and 37 ˚C in as described in section 1-2-1, 0.5 OD_600_ units of cell culture was mixed with 0.5 OD_600_ units of ^15^N spike culture, and the cells were pelleted by centrifugation at 14,000 rpm for 30 seconds. The cell pellet was washed with cold water for twice, before 13% TCA was added directly to the pellet, followed by vortex mixing and incubation for 12 hours at 4^o^C. The protein precipitate was pelleted by centrifugation at 14,000 rpm for 30 min at 4^o^C. The protein pellet was washed twice by adding 700 ul 10% TCA followed by centrifugation at 14,000 rpm. The final supernatant was removed, and the pellet was dried in a Speed-Vac concentrator, and then dissolved in 40 ul of fresh 100 mM ammonium bicarbonate and 5% acetonitrile (ACN). To break the disulfide bonds between cysteine residues, a 4 ul aliquot of 50 mM DTT was added to each sample, and the samples were incubated at 65 ˚C for 10 min. Thiol groups were blocked by the addition of 4 ul of 100 mM iodoacetamide (IAA) and incubation for 30 min at 30 ˚C in the dark. The protein was digested by the addition of 2 ul of 0.1 ug/ul modified sequence grade porcine trypsin (Promega, Co., Madison, WI) with incubation overnight at 37 ˚C. Undigested proteins were removed by adding 1/3 volume of 2% trifluoroacetic acid in 20% ACN with centrifugation, and the supernatant was transferred to a Pierce C-18 spin column (Thermo Fisher Scientific Inc., Rockford, IL) to remove salts and to concentrated the peptides. The sample was eluted from the column with 60 ul 70% ACN in 0.1% formic acid. The eluant was dried in a Speed-Vac concentrator and the peptides were resuspended in 10 ul 5% ACN in 0.1% formic acid.

Peptides were analyzed on a 5600+ Triple TOF mass spectrometer (T-TOF) coupled to Eksigent ekspert™ nanoLC 400 systems with ChromXP C18 (3 μm 120 A) column. The sample was injected via autosampler into the column with a trap-eluting mode using NanoLC Trap ChromXP C18 (3μm 120 A). NanoLC separation consisted of the following steps: (1) a linear gradient from 10% to 40% buffer B over 80 min, (2) column washing with 80% B for 20 min, and (3) column equilibration with 10% B for 30 min. The MS/MS data was collected with data-dependent acquisition mode on the T-TOF. The data was pre-processed according to Trans-Proteomic Pipeline^4^. Briefly, the raw data was transformed with MSConverter. Peptides were identified using X!Tandem^5^ searching against custom E. coli database (derived from UniProt organism 83333) and combined into spectral libraries using SpectraST^6^ before relative quantification in Massacre as previously described ^7,8^. The Massacre fitting results were filtered based on an in-house random forest filtering algorithm trained with peptide Massacre fits that were manually annotated as good or bad.

**1-2-3 SWATH mass spectrometry analysis of pre-50S/50S fractions**

For SWATH sample preparation, 50 pmol of ^15^N WT-70S was added to pre-50S fractions that were purified by sucrose gradient centrifugation. TCA was added to the spiked fraction to 13% final concentration to precipitate proteins. The tryptic peptide preparation was described in section 1-2-2. After resuspension, peptides were analyzed on the T-TOF coupled to an Agilent 1100 Series HPLC instrument with capillary flow electrospray (Agilent Technologies Inc., Santa Clara, CA). Peptides were injected using an autosampler onto an Agilent Zorbax SB C18 150 mm x 0.5 mm HPLC column, with the mobile phase 0.1% formic acid in HPLC grade H2O and acetonitrile with 0.1% formic acid. SWATH data-independent mass spectrometry data were collected using positive polarity over the m/z range of 300-1300. The mass spectral data were analyzed using Skyline ^9^, and the r-protein levels were quantified as previously described ^10^.

**1-2-4 Quantitative rRNA modification analysis of pre-50S/50S fractions**

The rRNA in pre-50S and 70S fractions were extracted with Direct-zol™ RNA MiniPrep Kits (Zymo) using the manufacturer’s instructions. The RNA MS/MS was performed as previously described ^11^. The rRNA isolated from mature ^15^N-ribosomes was added as an external standard. Purified ^14^N 23S from the test sample and ^15^N-RNA spike were mixed in approximately 1:1 molar ratio, heat-denatured, and digested with RNase T1 or A for 1 h at 55 °C in 25 mM ammonium acetate (pH = 6). Nucleolytic fragments were separated on an Xbridge C18 column (Waters) using buffer A (15 mM ammonium acetate, pH = 8.8) and buffer B (15 mM ammonium acetate, pH = 8.8 and 50% acetonitrile). HPLC separation consisted of the following steps: (1) isocratic elution with 1% buffer B for 5 min, (2) a linear gradient from 1% to 15% buffer B over 40 min, (3) column washing with 100% B for 25 min, and (4) column equilibration with 1% B for 30 min. Data were recorded over the 400–1700 m/z range on Agilent Q-TOF 6520 ESI. Pairs of the co-eluting ^14^N- and ^15^N-labeled fragments were identified using Pytheas ^12^. After the LC-MS peak profiles were extracted and fit using Agilent MassHunter.

**2 Workflow of Iterative classification with ab initio reconstruction in CryoSPARC**

A screenshot of the CryoSPARC work flow for reconstruction of intermediate density maps in the ∆*deaD* dataset is shown in Supplementary Fig. 20. The overall workflow is hierarchical, with the outputs of one job serving as the inputs to a subsequent series of jobs. As described in Methods, the cleaned particle stacks after RELION preprocessing were submitted to direct *ab initio* reconstruction, asking for 4 classes in job J62, shown in Supplementary Fig 19a. Classes 0 and 3 did not have interpretable particles, while classes 1 and 2 showed clear shapes of putative 50S intermediates (Supplementary Fig 19a-d). The 4 classes from J62 were analyzed by 2D classification (J168-171, Supplementary Fig 20e-h) to validate our picks for next round. These two stacks were subjected to the next round of *ab-initio* reconstruction, again with 4 classes, in jobs J63 and J64. In general, the subclassification was continued iteratively, as shown in Supplementary Fig 20, until one of two termination criteria was satisfied. As one example, the particle numbers in the subclassification job J130 were 1763, 32, 84 and 1742 for subclasses, 0,1,2, and 3, respectively, (green box in Supplementary Fig 20). Classes 0 and 3 were below the particle threshold of 2000, and termination was affected by 3D refinement in jobs J147 and J148, followed by *ab initio* reconstruction and 3D refinement. As a second example, after jobs J136 and J137, a 3D refinement of two subclasses yielded maps with differences below the 10 KDa threshold, so the particles were merged and subjected to *ab initio* reconstruction with 1 class (J283, red box in Supplementary Fig 20). Finally, for terminal species, *ab initio* reconstruction for 1 class (J283) was performed with 3D refinement (NEW) job (J304), before performing ThreeDFSC (job J326) for FSC calculation and resolution estimation (Purple box in Supplementary Fig 20). Class metadata for three datasets can be found in Supplementary Table 2 and the detailed information of iterative classification can be found in the Supplementary Data 1.

**3 Parameters in PCA-UMAP and method comparison**

This section discusses the optimization process of segmentation of a set of electron density maps using the dimensionality reduction methods of PCA and UMAP. The goal is to identify groups of voxels with similar behavior across the set of maps using a clustering method, and tuning the parameters of the PCA-UMAP to optimize performance and generation of interpretable segments. For scatter plot in this section, blocks are colored according to Figure 2 (the final optimal PCA-UMAP-HDBSCAN segmentation) in the main text, to illustrate the quality of block separation for subsequent cluster picking.

**3-1 Direct UMAP and different metrics**

The PCA-UMAP results described in Methods was compared to the direct application of UMAP on the 21 x 114,392 matrix without PCA transformation. Also, four metrics (Euclidean, Canberra, correlation and cosine) were applied in both the direct UMAP method or PCA-UMAP method, for comparison (Supplementary Fig. 21). We found that two UMAP-1,2 scatter plots are the same for direct UMAP and PCA-UMAP using the Euclidean metric. This may be expected since the PCA is a matrix factorization, which should not change the Euclidean distance if all PCs are used. (Supplementary Fig. 21b f). However, the blocks are better-defined in the PCA-UMAP compared to direct UMAP using the other three metrics (Canberra, correlation, and cosine). Among the eight different trials, PCA-UMAP with Canberra metric showed separated blocks with smallest average distance to cluster median with smallest variation (Supplementary Fig. 22).

Since, UMAP is inherently stochastic^13^, the stability of the algorithm was tested by running the same dataset for 100 times using direct UMAP and PCA-UMAP with Canberra metric with different seeds. The mean squared displacement (MSD) for each dot in the set of scatter plots is calculated. The MSD scatter plot (Supplementary Fig. 26) showed that the algorithm for PCA-UMAP with Canberra metric was very stable and a given block does not vary significantly between runs. In contrast direct application of UMAP exhibited highly variable positions of the blocks. (Supplementary Fig. 26b)

**3-2 PCA-UMAP with different number of principle components**

When UMAP was applied to PCA transformed features, we can control the number of features input to UMAP. Using a low number of PCs, the UMAP step was unable to separate clusters. With increasing number of PCs, the UMAP step stabilizes, such that there is little additional change when additional PCs are included. The Euclidean metric led to convergence with a smaller number of PCs, but the resulting distribution of blocks was elongated and tangled, making the subsequent extraction process difficult (Supplementary Fig. 23). Using the Canberra metric, gave a more separable distribution. (Supplementary Fig. 24). Both the Canberra and Euclidean metrics showed convergence with increasing PC number, suggesting that the higher PCs mostly represent the noise (Supplementary Fig. 6), having little influence on cluster formation. In practice, we use the full PCA reconstruction to reduce the possibility of subjective feature picking.

**3-3 Nearest neighbors in UMAP**

Supplementary Fig. 25 showed the tuning of the parameter for number of nearest neighbors in UMAP. We found if the number becomes too large, some clusters began to merge in to the large blocks. On the other hand, if the number of nearest neighbors is too small, the model captured some details that do not represent a whole block. We chose an intermediate value with 100 nearest neighbors, which roughly corresponds to the 3 KDa volume, corresponding approximately to a small RNA helix.

**4 Detailed ∆*deaD* block description**

**4-1 Assembly core**

The first block common to all maps in the dataset is termed the **Assembly core** as the origin of the assembly pathway. It consists of all helices in 23S Domain I, except H1, and four helices (H27-29, H31) in Domain II 23S rRNA, and the four early r-proteins, uL4, uL22, uL24 and uL29. uL24 binds to the most 5’ end is reported to be one of the two initiators for 50S formation in *in vitro* experiment ^14^. Although uL22 has contacts with all domains of 23S rRNA, the structured region of domain I rRNA (~600 nucleotides) is sufficient for uL22 to stably bind to 23S rRNA. This implies that uL22 can associate early with the nascent transcript. Interestingly, the Assembly core does not include H1 which is formed by the base pairing of 5’ and 3’ of the entire 23S rRNA, proximal to the RNase cleavage site that liberates the pre-50S particle from the primary rRNA transcript. This is the first evidence showing the early 50S assembly intermediates do not need H1 formation, which is strong evidence for the co-transcriptional block-wise assembly.

**4-2 bL20 Block**

The next largest block is the **bL20 Block,** which contains uL13, bL20, bL21, bL34 and most of the helices in domain II of 23S rRNA, with exception for four helices in assembly core, H33-35 and H42-44. The latter helices H42-44 correspond to the base for the right stalk where uL10/11 anchor which is not present in any of the intermediates in this data set. This block also contains a portion of the long helix 38 (H38bd) (Supplementary Fig. 11), while the remaining portion has more interaction with the central protuberance (H38cp).

**4-3 other blocks**

The **uL23 Block** contains uL23 and majority of domain III of 23S rRNA, except h55-59 and h106. The **uL3 block** contains uL3, bL17, bL19 and domain VI 23S of rRNA. Protein uL3 was the second initiator protein identified based on *in vitro* reconstitution experiments ^14^. The CP block contains 5S rRNA, the distal portion of H38 (H38cp), H81-88, and proteins L5, L15, L18, L25, L27, and L30. The uL2 block contains uL2 and part of domain IV rRNA. Other than these RNA-protein blocks, there are also three small blocks solely consisting of RNA helices, which are the H55-59, H33-35, H63, and uL1 base block.

**5 Dependency analysis**

**5-1 Redundant edge pruning**

Supplementary Fig. 28 showed criteria of removing an edge in the dependency network. Briefly, if block 2 depends on block 1, at the same time, block 3 depends on both block 1 and block 2. To simplify the model, we can remove edge from block 1 to block 3, without changing the fact of block 3 needs formation of both block 1 and block 2.

**6 rRNA modification**

**H33-35 formation and implication of early-stage rRNA modification**

Within the H33-35 block, H35 is modified at positions m^1^G (745), Ψ (746), and m^5^U (747). Quantitative RNA mass spectrometry shows that double methylation in the pre-50S fraction is over 95%, (Supplementary Fig. 4, Supplementary Data 2) while only about 40% of particles in the set of maps have H35 properly folded and docked. This evidence strongly suggests that the substrate for the corresponding enzymes RlmA and RlmC is a very immature pre50S particle. RlmA was shown to bind on free 23S^15^, however, substrate for RlmC is still unknown. Our results implied the early function of these methyl transferase in ribosome biogenesis.

**Supplementary Reference**

1 Charollais, J., Dreyfus, M. & Iost, I. CsdA, a cold-shock RNA helicase from Escherichia coli, is involved in the biogenesis of 50S ribosomal subunit. *Nucleic Acids Res* **32**, 2751-2759 (2004). <https://doi.org:10.1093/nar/gkh603>

2 Ojha, S. & Jain, C. Dual-level autoregulation of the E. coli DeaD RNA helicase via mRNA stability and Rho-dependent transcription termination. *RNA* **26**, 1160-1169 (2020). <https://doi.org:10.1261/rna.074112.119>

3 Vakulskas, C. A. *et al.* Global effects of the DEAD-box RNA helicase DeaD (CsdA) on gene expression over a broad range of temperatures. *Mol Microbiol* **92**, 945-958 (2014). <https://doi.org:10.1111/mmi.12606>

4 Deutsch, E. W. *et al.* A guided tour of the Trans-Proteomic Pipeline. *Proteomics* **10**, 1150-1159 (2010). <https://doi.org:10.1002/pmic.200900375>

5 Craig, R. & Beavis, R. C. TANDEM: matching proteins with tandem mass spectra. *Bioinformatics* **20**, 1466-1467 (2004). <https://doi.org:10.1093/bioinformatics/bth092>

6 Lam, H. *et al.* Building consensus spectral libraries for peptide identification in proteomics. *Nat Methods* **5**, 873-875 (2008). <https://doi.org:10.1038/nmeth.1254>

7 Sperling, E., Bunner, A. E., Sykes, M. T. & Williamson, J. R. Quantitative analysis of isotope distributions in proteomic mass spectrometry using least-squares Fourier transform convolution. *Anal Chem* **80**, 4906-4917 (2008). <https://doi.org:10.1021/ac800080v>

8 Sykes, M. T., Sperling, E., Chen, S. S. & Williamson, J. R. Quantitation of the ribosomal protein autoregulatory network using mass spectrometry. *Anal Chem* **82**, 5038-5045 (2010). <https://doi.org:10.1021/ac9028664>

9 MacLean, B. *et al.* Skyline: an open source document editor for creating and analyzing targeted proteomics experiments. *Bioinformatics* **26**, 966–968 (2010). <https://doi.org:10.1093/bioinformatics/btq054>

10 Davis, J. H. *et al.* Modular Assembly of the Bacterial Large Ribosomal Subunit. *Cell* **167**, 1610-1622 e1615 (2016). <https://doi.org:10.1016/j.cell.2016.11.020>

11 Popova, A. M. & Williamson, J. R. Quantitative analysis of rRNA modifications using stable isotope labeling and mass spectrometry. *J Am Chem Soc* **136**, 2058-2069 (2014). <https://doi.org:10.1021/ja412084b>

12 D'Ascenzo, L. *et al.* Pytheas: a software package for the automated analysis of RNA sequences and modifications via tandem mass spectrometry. *Nat Commun* **13**, 2424 (2022). <https://doi.org:10.1038/s41467-022-30057-5>

13 McInnes, L., Healy, J. & Melville, J. Umap: Uniform manifold approximation and projection for dimension reduction. *arXiv preprint arXiv:1802.03426* (2018).

14 Nowotny, V. & Nierhaus, K. H. Initiator proteins for the assembly of the 50S subunit from Escherichia coli ribosomes. *Proc Natl Acad Sci U S A* **79**, 7238-7242 (1982). <https://doi.org:10.1073/pnas.79.23.7238>

15 Hansen, L. H., Kirpekar, F. & Douthwaite, S. Recognition of nucleotide G745 in 23 S ribosomal RNA by the rrmA methyltransferase. *J Mol Biol* **310**, 1001-1010 (2001). <https://doi.org:10.1006/jmbi.2001.4836>
