# Supplementary material for: Assembly landscape for the bacterial large ribosomal subunit
Source: Nat Commun. 2023 Aug 26;14:5220. doi: 10.1038/s41467-023-40859-w (PMC10460392; doi:10.1038/s41467-023-40859-w)
Supplement: Supplementary file 3 — Description of Additional Supplementary Files [file 41467_2023_40859_MOESM3_ESM.pdf]

Description of Additional Supplementary Files

File Name: Supplementary Data 1
Description: Job log for Iterative ab-initio subclassification and merge jobs in three CryoSPARC projects (Δ*deaD*, Δ*srmB* and bL17-depletion) are described. “jid” gives the unique local job ID. The “subclassification/merge” column indicates either subclassification of the particles using ab-initio reconstruction, or the merging of classes for similar maps. For subclassification jobs, particles from one interpretable class from the parental job are subjected to ab-initio reconstruction, specifying 4 classes, resulting in 1-4 interpretable child classes. The particle numbers for all 4 classes are shown in the following particle_num_c1-4 columns. For merging jobs all particles from the parental jobs are collected, perform ab-initio reconstruction for a single class. The output particle number “particle_num_c1” is equal to input “particle_num”.

File Name: Supplementary Data 2
Description: **Sheet 1:** Data for Supplementary figure 3.
SWATH data for 50S and pre50S peaks. 1-3 detected peptides are displayed in the 50S and pre50S column for each protein. The average and standard deviation are calculated in the following column.
Sheet 2: Data for Supplementary figure 4.
RNA Mass spectrometry for modification analysis. The modified RNA oligos in 23S rRNA were observed using either RNase A or T1 digestion indicated in the “RNase” column, with observed modification sites in “mods” column. The identified sequence is in “sequence mods column”. The normalized ratio for pre50S, 50S and 70S against N15 70S spike are in following columns. Experiments for pre50S are duplicated.
Sheet 3:
Data for Supplementary figure 2b.
Whole cell proteomics for BW25113 *E. coli* at 19 ˚C (LB, ^{14}N) and 37 ˚C (M9, ^{15}N) at steady state. Output from the massacre analysis is shown for identified $^{14}N/^{15}N$ peptide pairs including molecular weight (“mw”), charge state (“isoz_charge”), and amplitudes of the massacre fit for ^{14}N and ^{15}N (“AMP_WT_19C_N14” vs. “AMP_WT_37C_N15”), etc. are included.
Sheet 4:
Data for Supplementary figure 2a.
Ribosomal protein in whole cell lysate of BW25113 (37 ˚C, LB, ^{14}N), Δ*deaD* (19/37 ˚C, LB, ^{14}N) compared to BW25113 (37 ˚C, M9, ^{15}N). The output of massacre amplitudes for each peptide ^{14}N or ^{15}N was normalized using all identified peptides from ^{14}N or ^{15}N respectively. The median ratio for a protein represents median of normalized ^{14}N amplitude/normalized ^{15}N amplitude for peptides.
