# Supplementary material for: Assembly landscape for the bacterial large ribosomal subunit
Source: Nat Commun. 2023 Aug 26;14:5220. doi: 10.1038/s41467-023-40859-w (PMC10460392; doi:10.1038/s41467-023-40859-w)
Supplement: Supplementary file 6 — Reporting Summary [file 41467_2023_40859_MOESM6_ESM.pdf]

nature portfolio

Corresponding author(s): Dr. James Williamson

Last updated by author(s): Jun 30, 2023

Reporting Summary

Nature Portfolio wishes to improve the reproducibility of the work that we publish. This form provides structure for consistency and transparency in reporting. For further information on Nature Portfolio policies, see our Editorial Policies and the Editorial Policy Checklist.

Statistics

For all statistical analyses, confirm that the following items are present in the figure legend, table legend, main text, or Methods section.

n/a	Confirmed	
☐	☒	The exact sample size (*n*) for each experimental group/condition, given as a discrete number and unit of measurement
☒	☐	A statement on whether measurements were taken from distinct samples or whether the same sample was measured repeatedly
☒	☐	The statistical test(s) used AND whether they are one- or two-sided<br>*Only common tests should be described solely by name; describe more complex techniques in the Methods section.*
☒	☐	A description of all covariates tested
☒	☐	A description of any assumptions or corrections, such as tests of normality and adjustment for multiple comparisons
☐	☒	A full description of the statistical parameters including central tendency (e.g. means) or other basic estimates (e.g. regression coefficient) AND variation (e.g. standard deviation) or associated estimates of uncertainty (e.g. confidence intervals)
☒	☐	For null hypothesis testing, the test statistic (e.g. *F*, *t*, *r*) with confidence intervals, effect sizes, degrees of freedom and *P* value noted<br>*Give P values as exact values whenever suitable.*
☒	☐	For Bayesian analysis, information on the choice of priors and Markov chain Monte Carlo settings
☒	☐	For hierarchical and complex designs, identification of the appropriate level for tests and full reporting of outcomes
☒	☐	Estimates of effect sizes (e.g. Cohen's *d*, Pearson's *r*), indicating how they were calculated

Our web collection on statistics for biologists contains articles on many of the points above.

Software and code

Policy information about availability of computer code

Data collection	All single particle CryoEM data is collected using Thermo Fisher Scientific Titan Krios electron microscope operating at 300k eV equipped with a Gatan K2 Summit detector using the Leginon software which discussed in detail in the Method session. The data acquisition was assisted with Leginon.
Data analysis	Data pre-processing, including motion correction and CTF estimation, was performed within the Appion pipeline. Frames were aligned using MotionCor2, and the contrast transfer function (CTF) for all micrographs was estimated with CTFFind4.1. The aligned frame sums were then imported into RELION3. Particles from the dataset were picked with auto picking in RELION3, roughly filtered with 2D classification and 3D classification in RELION3. Particle stacks were exported and analyzed in CryoSPARC3 (discussed in details in Method and SI). Reconstructed maps were aligned and resampled in ChimeraX-1.2.5. All structure figures are prepared with ChimeraX-1.2.5.<br>Following data analysis including segmentation, occupancy, quadrant and dependency analyses are performed using python/3.6 using the following packages: numpy-1.20.1, pandas-1.2.4, mrcfile-1.3.0, umap-0.5.1, sklearn-0.24.1, networkx-2.5, hdbscan and pickle.<br><br>SWATH data for ribosomal protein composition was analyzed with Skyline. Whole cell proteomics are analyzed by X!tandem, SpectraST and Massacre software. RNA mass spectrometry data was analyzed by Pytheas and Agilent MassHunter.

For manuscripts utilizing custom algorithms or software that are central to the research but not yet described in published literature, software must be made available to editors and reviewers. We strongly encourage code deposition in a community repository (e.g. GitHub). See the Nature Portfolio guidelines for submitting code & software for further information.

Data

Policy information about availability of data

All manuscripts must include a data availability statement. This statement should provide the following information, where applicable:

- Accession codes, unique identifiers, or web links for publicly available datasets
- A description of any restrictions on data availability
- For clinical datasets or third party data, please ensure that the statement adheres to our policy

The doubling time table is in Supplementary Table 1.
We used PDB:4ybb as the atomic model reference for matured E. coli ribosome
64 EM density maps were deposited into EMDB, details are listed in Supplementary Table 2. Here are the EMDB IDs:
EMD-40517, EMD-40519, EMD-40520, EMD-40524, EMD-40526, EMD-40528, EMD-40530, EMD-40532, EMD-40534, EMD-40536, EMD-40538, EMD-40540, EMD-40542, EMD-40544, EMD-40546, EMD-40548, EMD-40550, EMD-40552, EMD-40555, EMD-40551, EMD-40549, EMD-40309, EMD-40311, EMD-40313, EMD-40511, EMD-40314, EMD-40315, EMD-40317, EMD-40319, EMD-40321, EMD-40323, EMD-40327, EMD-40329, EMD-40331, EMD-40333, EMD-40512, EMD-40514, EMD-40516, EMD-40518, EMD-40521, EMD-40523, EMD-40525, EMD-40527, EMD-40529, EMD-40531, EMD-40533, EMD-40535, EMD-40537, EMD-40539, EMD-40541, EMD-40543, EMD-40545, EMD-40547, EMD-40316, EMD-40318, EMD-40320, EMD-40322, EMD-40324, EMD-40325, EMD-40328, EMD-40330 ,EMD-40332 ,EMD-40513 ,EMD-40515
CryoSPARC job description is included Supplementary Data 1.
Mass spectrometry data are included in Supplementary Data 2.

Human research participants

Policy information about studies involving human research participants and Sex and Gender in Research.

Reporting on sex and gender	Not Applicable
Population characteristics	Not Applicable
Recruitment	Not Applicable
Ethics oversight	Not Applicable

Note that full information on the approval of the study protocol must also be provided in the manuscript.

Field-specific reporting

Please select the one below that is the best fit for your research. If you are not sure, read the appropriate sections before making your selection.

☒ Life sciences ☐ Behavioural & social sciences ☐ Ecological, evolutionary & environmental sciences

For a reference copy of the document with all sections, see nature.com/documents/nr-reporting-summary-flat.pdf

Life sciences study design

All studies must disclose on these points even when the disclosure is negative.

Sample size	We did not calculate the sample size. 1,031 micrographs were collected for ΔdeaD dataset. 21 maps were reconstructed with sufficient resolution.<br>123,804 and 273,620 particles for ΔsrmB and bL17-depletion dataset were extracted from published micrographs, respectively. The result intermediate map also achieved sufficient resolution.
Data exclusions	Uninterpretable reconstructions are excluded, discussed in details in Methods and SI.
Replication	The CryoEM study is not repeated as each structure are averaged from many particles and achieved reasonable resolution.
Randomization	Each particle during the data acquisition was a independent observation, and we have more than 0.1 M particles for each dataset, from which we have achieved sufficient resolution for our purposes. Also, the data process include the randomization during the classification step. PCA-UMAP has been run 100 times on the same dataset. The algorithm is stable and similar results were achieved every time, discussed in SI.
Blinding	No blinding was required for the reported experiments, and we did not do blinding.

Reporting for specific materials, systems and methods

We require information from authors about some types of materials, experimental systems and methods used in many studies. Here, indicate whether each material, system or method listed is relevant to your study. If you are not sure if a list item applies to your research, read the appropriate section before selecting a response.

Materials & experimental systems

n/a	Involved in the study	
☒	☐	Antibodies
☒	☐	Eukaryotic cell lines
☒	☐	Palaeontology and archaeology
☒	☐	Animals and other organisms
☒	☐	Clinical data
☒	☐	Dual use research of concern

Methods

n/a	Involved in the study	
☒	☐	ChIP-seq
☒	☐	Flow cytometry
☒	☐	MRI-based neuroimaging
